# Prenatal social support in low-risk pregnancy shapes placental epigenome

**DOI:** 10.1186/s12916-022-02701-w

**Published:** 2023-01-08

**Authors:** Markos Tesfaye, Jing Wu, Richard J. Biedrzycki, Katherine L. Grantz, Paule Joseph, Fasil Tekola-Ayele

**Affiliations:** 1 Section of Sensory Science and Metabolism (SenSMet), National Institute on Alcohol Abuse and Alcoholism & National Institute of Nursing Research, National Institutes of Health, Bethesda, MD USA; 2 Department of Psychiatry, St. Paul’s Hospital Millennium Medical College, Addis Ababa, Ethiopia; 3 Glotech, Inc., contractor for Division of Population Health Research, Division of Intramural Research, Eunice Kennedy Shriver National Institute of Child Health and Human Development, National Institutes of Health, Bethesda, MD USA; 4 Epidemiology Branch, Division of Population Health Research, Division of Intramural Research, Eunice Kennedy Shriver National Institute of Child Health and Human Development, National Institutes of Health, MD Bethesda, USA

**Keywords:** Social support, Placenta, DNA methylation, Neurodevelopment, Energy metabolism, Pregnancy

## Abstract

**Background:**

Poor social support during pregnancy has been linked to inflammation and adverse pregnancy and childhood health outcomes. Placental epigenetic alterations may underlie these links but are still unknown in humans.

**Methods:**

In a cohort of low-risk pregnant women (*n* = 301) from diverse ethnic backgrounds, social support was measured using the ENRICHD Social Support Inventory (ESSI) during the first trimester. Placental samples collected at delivery were analyzed for DNA methylation and gene expression using Illumina 450K Beadchip Array and RNA-seq, respectively. We examined association between maternal prenatal social support and DNA methylation in placenta. Associated cytosine-(phosphate)-guanine sites (CpGs) were further assessed for correlation with nearby gene expression in placenta.

**Results:**

The mean age (SD) of the women was 27.7 (5.3) years. The median (interquartile range) of ESSI scores was 24 (22–25). Prenatal social support was significantly associated with methylation level at seven CpGs (*P*_FDR_ < 0.05). The methylation levels at two of the seven CpGs correlated with placental expression of *VGF* and *ILVBL* (*P*_FDR_ < 0.05), genes known to be involved in neurodevelopment and energy metabolism. The genes annotated with the top 100 CpGs were enriched for pathways related to fetal growth, coagulation system, energy metabolism, and neurodevelopment. Sex-stratified analysis identified additional significant associations at nine CpGs in male-bearing pregnancies and 35 CpGs in female-bearing pregnancies.

**Conclusions:**

The findings suggest that prenatal social support is linked to placental DNA methylation changes in a low-stress setting, including fetal sex-dependent epigenetic changes. Given the relevance of some of these changes in fetal neurodevelopmental outcomes, the findings signal important methylation targets for future research on molecular mechanisms of effect of the broader social environment on pregnancy and fetal outcomes.

**Trial registration:**

NCT00912132 (ClinicalTrials.gov).

**Supplementary Information:**

The online version contains supplementary material available at 10.1186/s12916-022-02701-w.

## Background

Social support promotes mental and physical health in low stress environments [1, 2] and buffers the effects of stress in high stress environments [3, 4]. Maternal resilience factors such as prenatal social support have been linked to higher leukocyte telomere length in newborns [5] and lower adiposity during infancy [6]. Moreover, poor social support in early childhood may influence health outcomes later in life [7]. However, little is known about the biological mechanisms that underlie the relationship between prenatal social support and subsequent health outcomes.

The placenta undergoes dynamic DNA methylation changes throughout pregnancy in response to biological and environmental factors to provide an optimal environment for fetal development [8, 9]. Emerging evidence suggests that epigenetics may partly explain the link between prenatal psychosocial factors, such as maternal stress and depression, and child health outcomes [10]. Therefore, it is possible that social support during pregnancy may influence fetal development and long-term health outcomes by altering the placental epigenome. However, there is no published study on the association between social support and genome-wide DNA methylation of human placenta. Prenatal social support in humans has been associated with DNA methylation in maternal blood [11], and social rank in primates has been associated with placental DNA methylation [12]. Low social support has been linked to inflammation [13], and quality of prenatal social support has been linked to inflammation during pregnancy and early infancy [14, 15]. Therefore, identifying placental DNA methylation changes associated with prenatal social support in low-risk pregnancies may shed light on the molecular mechanisms underlying the effects of social support on fetal development, crucial information for developing interventions to promote fetal development and long-term health outcomes.

Using the *Eunice Kennedy Shriver* National Institute of Child Health and Human Development (NICHD) Fetal Growth Studies (FGS) cohort data [16], we investigated the association between maternal social support during pregnancy and genome-wide DNA methylation in placenta at delivery. Given accumulating evidence on sex differences in placental methylation [17–20] and placental response to adverse prenatal environments [10, 21, 22], we also investigated the association separately in male and female fetuses. For cytosine-(phosphate)-guanine sites (CpGs) found to be significantly associated with social support, we examined whether methylation of CpGs was associated with expression of nearby genes in placenta.

## Methods

### Setting and subjects

We used data from the *Eunice Kennedy Shriver* NICHD FGS – Singletons. Among the total 2802 participants, 312 had placenta samples collected at delivery. Participants who provided placenta and those who did not provide placenta did not have significant differences in maternal age, fetal sex, job status, educational status, social support, or perceived stress scores (Additional file 1: Table S1). The study was approved by the Institutional Review Boards of NICHD and all respective participating clinical sites. All participants provided informed consent at enrollment into the study.

The participants were low risk pregnant women enrolled at gestational ages of 8 to 13 weeks from 12 clinics in the USA during the period between July 2009 and January 2013. The inclusion criteria were age 18–40 years, viable singleton pregnancy, and planning to give birth at the participating health facilities. Exclusion criteria were previous history of poor obstetric outcomes, pre-existing chronic medical and psychiatric conditions, smoking in the previous 6 months or use of illicit drugs during the previous 12 months, and consumption of ≥ 1 alcohol drink daily [16].

### Main exposure variable

Maternal social support was assessed at enrollment using the self-report Enhancing Recovery in Coronary Heart Disease Social Support Instrument (ESSI) [23]. ESSI uses seven items for assessing the degree of social support an individual has. The higher total scores higher scores indicate greater degree of social support.

### Covariates

Data on maternal age, parity, education, maternal job status, pre-pregnancy BMI, self-identified race/ethnicity, gestational age at delivery, and fetal sex were obtained through interviews and from medical records as described elsewhere [16]. Perceived stress was measured using the self-report ten-item Cohen’s Perceived Stress Scale (PSS-10). A higher PSS-10 score indicates greater level of perceived stress [24].

### Placenta sample collection and DNA methylation quantification

Placentas obtained at delivery were rinsed with sterile saline, pat dried with paper towel, and had nonadherent clots removed. The placental membrane and umbilical cord were trimmed before biopsies were taken. Four biopsies measuring 0.5 cm × 0.5 cm × 0.5 cm were collected directly below the fetal surface of each placenta within 1 h of delivery. The samples were placed in RNALater and frozen at – 80 °C for molecular analysis. The placental biopsy samples were processed at the Columbia University Irving Medical Center as described previously [25]. DNA was extracted from the samples and assayed using the Illumina Infinium Human Methylation450 Beadchip (Illumina Inc., San Diego, CA) array. A total of 301 placental samples that passed quality control were included in the analysis [26]. Eleven samples were excluded because they were outliers from the distribution of genetic clusters of the sample (*n* = 6), genotype sex mismatch between fetus and placenta (*n* = 4), and mismatch of sample identifiers (*n* = 1).

Standard Illumina protocols were followed for background correction, normalization to internal control probes, and quantile normalization. The Illumina 450k array’s plating scheme was adjusted according to the assay’s internal QC design. The GenomeStudio QC standard was implemented during data preprocessing, and the internal probes have been used for background correction, dye bias correction, normalization, probe-design bias correction, and an offset for Infinium I and II probe intensity. The assay quality controls comprised of controls for measuring staining sensitivity and controls for testing efficiency of bisulfite conversion. Bisulfite modification was performed using the EZ Methylation kit (Zymo Research, CA). Bisulfite-converted sequences without CpGs served as negative controls; the mean of the negative control probes was used as the system background. The resulting intensity files were processed with Illumina’s Genome Studio which generated average beta values for each CpG site (i.e., the fraction of methylated sites per sample by calculating the ratio of methylated and unmethylated fluorescent signals) and detection *P*-values which characterized the chance that the target sequence signal was distinguishable from the negative controls. The method was corrected for probe design bias in the Illumina Infinium Human Methylation450 BeadChip and achieved between-sample normalization. Normalization was performed using the modified Beta Mixture Quantile dilation (BMIQ) method to correct the probe design bias in the Illumina Infinium Human Methylation450 BeadChip and achieve between-sample normalization [27].

Missing CpGs were imputed by the k-nearest neighbors method, setting *k* = 10. Beta values with an associated detection *P* ≥ 0.05 were set to missing. Probes with mean detection *P* ≥ 0.05 (*n* = 36), cross-reactive (*n* = 24,491), non-autosomal (*n* = 14,589), and CpG sites within 20 bp from a known single nucleotide polymorphism (SNP) (*n* = 37,360) were removed [20]. Consequently, methylation data for 409,101 were obtained for analysis. We transformed the beta values to *M* value scale before analysis as recommended using the formula *M* = log2(Beta/(1-Beta)) [28].

#### RNA extraction and quantification

RNA from 80 placenta samples was isolated using TRIZOL reagent (Invitrogen, MA, USA). The mRNA libraries were sequenced on an Illumina HiSeq2000 machine with 100 bp paired-end reads as described elsewhere [25]. Data from 75 participants who had both DNA methylation and RNA-seq data were used for the methylation and gene expression association tests.

### Statistical analysis

#### Association between CpG sites and social support

We performed epigenome-wide analyses using multiple linear regression models with the DNA methylation CpG site as response variable on the *M* value scale and maternal social support scores as predictor. We also performed similar analyses by subgroups based on fetal sex. All regression analysis models were adjusted for maternal age, parity, education, maternal job status, pre-pregnancy BMI, self-identified race/ethnicity, gestational age at delivery, fetal sex, maternal perceived stress scores measured at recruitment, 10 genetic principal components computed from genome-wide autosomal SNP genotypes of placenta from HumanOmni2.5 Beadchips (Illumina Inc., San Diego, CA) to adjust for population structure, three methylation-based principal components, methylation sample plate, and components based on putative cell-mixture estimates using surrogate variable analysis (SVA) to account for confounding by variation in cell composition [29]. In sensitivity analyses, linear regression models were additionally adjusted for cell composition variables created using methods developed by Yuan et al. [30]. Further sensitivity analysis was performed to assess whether the significant associations remain in a statistical model that did not include maternal sociodemographic factors (i.e., by excluding maternal age, parity, education, maternal job status, pre-pregnancy BMI, self-identified race/ethnicity, and gestational age at delivery from list of adjusted covariates). We assessed the direction of association and correlation of methylation fold-changes (logFC) between the fully adjusted model and the model without sociodemographic covariates.

Differentially methylated CpG sites were mapped to genes within 250kb using R/Bioconductor package (IlluminaHumanMethylation450kmanifest) with a reference consisting of all genes present in the Illumina 450k platform. *P*-values were corrected for false discovery rate (FDR) using the Benjamini-Hochberg method. *P*-values were further corrected for genomic inflation (*λ*) by applying a Bayesian method in R/Bioconductor package (BACON) [31]. Quantile-Quantile (QQ) plots were generated for the regression models before and after BACON correction. The QQ plots do not exhibit significant inflation of the *p*-values with *λ* = 1.0, *λ* = 1.03, and *λ* = 0.97 after BACON correction for the overall, male-specific, and female-specific results, respectively (Additional file 1: Figure S1–S3). For sex-stratified analyses, we followed the approach described by Randall et al. which implements Welch’s *t*-test [32] to categorize the associations into one of three groups: (i) concordant effect direction (CED) defined, for effects sizes in the same direction, as association that is significant at *P*_FDR_ < 0.05 in one fetal sex and at least nominally significant in the other fetal sex; (ii) single sex effect (SSE) when significant association is present in one fetal sex (*P*_FDR_ < 0.05) and no association observed in the other fetal sex; or (iii) opposite effect direction (OED) defined, for effect sizes in opposite direction, as association that is significant in one fetal sex (*P*_FDR_ < 0.05) and at least nominally significant in the other fetal sex. Post hoc statistical power analysis was performed using two-tailed tests assuming probability of error (*α*) = 0.05 and demonstrated that the study power was ≥ 90% for detecting the effect sizes of 82% of the CpGs found to be associated with social support in the overall as well as sex-stratified analyses (Additional file 1: Figure S4).

We employed the R package *dmrff* to identify differentially methylated regions (DMR) in placenta associated with maternal social support at 5% FDR [33]. A DMR was defined to have a maximum length of 500 base pairs harboring a set of CpGs with EWAS *P* < 0.05 and identical effect direction.

#### Association between DNA methylation and gene expression

We analyzed association between DNA methylation at differentially methylated CpG sites and placental expression of protein-coding genes located within 250kb up- and downstream from the CpG sites using linear regression. Correlations between expression of the genes and social support scores were assessed using Pearson’s correlation test.

#### Functional annotations and regulatory enrichment

We examined whether genetic variants influence DNA methylation levels of the CpGs associated with social support. For this, we explored the CpGs in the list of known placental methylation quantitative trait loci (mQTLs) [25].

Using eFORGE version 2.0 [34], we examined enrichment and depletion of the CpGs significantly associated with social support (*P*_FDR_ < 0.05) for tissue or cell-type specific regulatory features. The CpGs identified in the total, male, and female samples were submitted to eFORGE and evaluated separately for overlap with DNase I hypersensitive sites, all 15-state chromatin marks, and all five H3 histone marks (i.e., H3K27me3, H3K4me1, H3K4me3, H3K36me3, H3K9me3).

#### Pathway enrichment analysis

We examined the biological functions of genes annotated to the top 100 CpG sites associated with social support using Ingenuity Pathway Analysis (IPA, Qiagen, Redwood City, CA, USA), separately for the overall and sex-stratified analysis results. Enriched biological pathways which contain at least two of the query genes and with *P*-values less than 0.05 were considered significantly enriched.

## Results

The characteristics of the 301 participants have been described previously [35]. Briefly, the mean age (SD) of the women was 27.7 (5.3) years; 50.5% of the fetuses were male. The median (interquartile range, IQR) of ESSI scores was 24 (22–25). The ESSI scores were relatively low with the 75th centile being equivalent to the 25th centile of the ESSI tool development study where the participants were individuals who had recent myocardial infarction [36]. The median (IQR) perceived stress score was 11 (6–14) as described elsewhere [21], which is lower than the corresponding figures in a US cohort of pregnant women during the first trimester [37] and normative data of Swedish women 14 (10–19) [38]. ESSI scores were positively correlated with having high school or higher educational status (*r* = 0.16, *P* = 0.007) and being employed (*r* = 0.12, *P* = 0.046) and inversely correlated with higher PSS-10 scores (*r* = − 0.34, *P* = 2.2 × 10^−9^).

### Maternal social support and DNA methylation in placenta at delivery

Higher maternal social support during the first trimester of pregnancy was associated with higher methylation at seven CpGs (located within/near genes *HAUS3*, *ARHGEF7*, *VGF*, *FAM210B*, *SBF1*, *ILVBL* and *EIF3F*) (BACON-corrected *P*_FDR_ ≤ 0.05). Most of these CpGs were either in promoter regions or gene bodies of the annotated genes. Also, the majority (6/7) loci were located in CpG islands (Table 1). In sensitivity analysis using a model additionally adjusted for cell composition variables, the methylation at these CpGs was associated with social support at *P*_FDR_ < 0.001 (Additional file 1: Table S2). In sensitivity analysis without maternal sociodemographic factors, all seven association directions remained the same and the correlation in logFC between the fully adjusted model and the model without sociodemographic covariates was perfect (*r* = 1, *P* = 2.8 × 10^−6^) (Additional file 1: Table S2).

**Table 1:** Methylation sites in placenta associated with level of social support during pregnancy (*n* = 301)

CpG	Gene	Chr: position	Relation to Gene	Relation to Island	Mean methylation Beta (SD)	Methylation LogFC ± S.E.	*P*-value^a^	*P*_FDR_
cg14806252	*HAUS3*	4:2244001	TSS200	Island	0.008 (0.005)	0.22 ± 0.04	4.6 × 10^−8^	0.019
cg01924481	*SBF1*	22:50898563	Body	Island	0.865 (0.020)	0.02 ± 0.003	1.3 × 10^−7^	0.021
cg11364468	*VGF*	7:100807505	Body	Island	0.011 (0.006)	0.11 ± 0.02	1.5 × 10^−7^	0.021
cg00549575	*EIF3F*	11:8008752	TSS200	N_Shore	0.040 (0.016)	0.08 ± 0.02	3.3 × 10^−7^	0.030
cg19499754	*FAM210B*	20:54919155		Island	0.029 (0.013)	0.11 ± 0.02	3.7 × 10^−7^	0.030
cg16763895	*ILVBL*	19:15235973	5'UTR	Island	0.030 (0.010)	0.07 ± 0.01	6.0 × 10^−7^	0.041
cg02672368	*ARHGEF7*	13:111805930	Body; TSS200	Island	0.017 (0.008)	0.13 ± 0.03	8.4 × 10^−7^	0.049

^a^Adjusted for maternal age, race/ethnicity, pre-pregnancy BMI, education, job status, gestational age, parity, fetal sex, perceived stress, methylation principal components, genotype principal components, and surrogate variable

*CpG* cytosine-(phosphate)-guanine site, *FDR* false discovery rate, *LogFC* logarithm of fold change, *S.E* standard error, *SD* standard deviation

### Maternal social support and fetal sex-specific DNA methylation in placenta

**Fig. 1:**
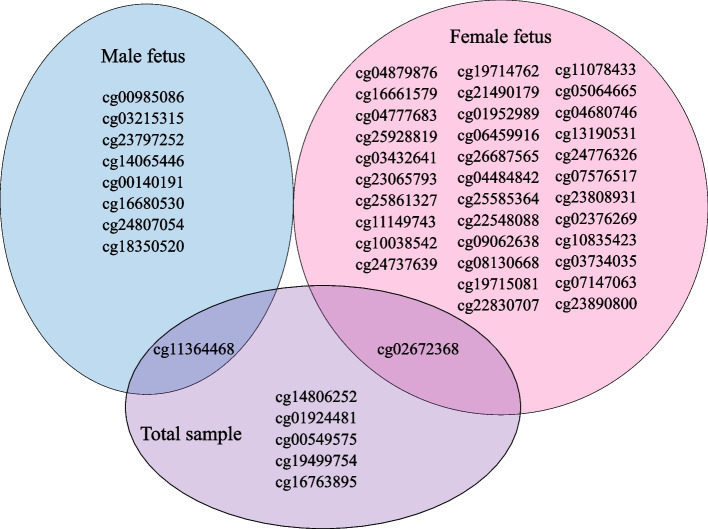
Placental methylation sites associated with social support during pregnancy by sex of the fetus. All models are adjusted to maternal age, ethnicity, pre-pregnancy body mass index (BMI), education, job status, gestational age, parity, perceived stress, methylation principal components (PCs), genotype PCs, and surrogate variable. The model for the total sample is additionally adjusted for sex of the fetus

In analyses grouped by fetal sex, maternal social support was associated with higher methylation at nine CpGs in males (all exhibiting SSE, *P*_FDR_ < 0.05) and with higher methylation at 32 CpGs and lower methylation at three CpGs in females (32 exhibiting SSE, 2 exhibiting OED, *P*_FDR_ < 0.05) (Table 2; Additional file 1: Tables S3 & S4). In sex-stratified sensitivity analyses where the model additionally included cell composition variables, methylation at the 44 CpGs were associated with social support at *P*_FDR_ < 0.001 (Additional file 1: Tables S5 & S6). In sensitivity analysis without maternal sociodemographic factors, all sex-specific association directions remained the same and the correlation in logFC between the fully adjusted model and the model without sociodemographic covariates was nearly perfect (male *r* = 0.99, *P* = 1.3 × 10^−6^; female *r* = 0.99, *P* < 2.2 × 10^−16^) (Additional file 1: Tables S5 & S6). Only two social support-associated CpGs in the overall sample, cg11364468 [*VGF*] and cg02672368 [*ARHGEF7*], were significant in male- and female-stratified analysis, respectively (Fig. 1). None of the CpGs associated with social support demonstrated concordant effects by fetal sex (Table 2).

**Table 2:** Comparison of effect sizes of social support-associated methylation sites between male fetus- and female fetus-bearing pregnancies

CpG	Gene	Total Sample ^a^			Male Fetus ^a^			Female Fetus ^a^			Sex difference statistics^b^	
		Methylation LogFC ±S.E.	*P*_FDR_	Mean methylation Beta (SD)	Methylation LogFC ± S.E	*P*_FDR_	Mean methylation Beta (SD)	Methylation LogFC ± S.E	*P*_FDR_	Mean methylation Beta (SD)	Welch’s *t* -test	*P*_FDR_
Associations identified in the overall sample												
cg14806252	*HAUS3*	0.22 ± 0.04	0.019	0.008 (0.005)	0.19 ± 0.06	0.655	0.008 (0.005)	0.23 ± 0.06	0.117	0.008 (0.005)	-	-
cg01924481	*SBF1*	0.02 ± 0.003	0.021	0.865 (0.020)	0.01 ± 0.005	0.959	0.866 (0.019)	0.02 ± 0.005	0.158	0.865 (0.021)	-	-
cg11364468	*VGF*	0.11 ± 0.02	0.021	0.011 (0.006)	0.21 ± 0.04	0.001	0.011 (0.006)	0.004 ± 0.02	0.999	0.011 (0.006)	4.61	1.24 × 10^−5^
cg00549575	*EIF3F*	0.08 ± 0.02	0.030	0.040 (0.016)	0.13 ± 0.03	0.066	0.040 (0.015)	0.02 ± 0.01	0.965	0.041 (0.016)	-	-
cg19499754	*FAM210B*	0.11 ± 0.02	0.030	0.029 (0.013)	0.10 ± 0.03	0.577	0.028 (0.013)	0.11 ± 0.03	0.344	0.030 (0.013)	-	-
cg16763895	*ILVBL*	0.07 ± 0.01	0.041	0.030 (0.010)	0.11 ± 0.03	0.147	0.030 (0.011)	0.005 ± 0.01	0.998	0.031 (0.010)	-	-
cg02672368	*ARHGEF7*	0.13 ± 0.03	0.049	0.017(0.008)	0.05 ± 0.03	0.961	0.017 (0.008)	0.21 ± 0.04	0.010	0.017 (0.008)	− 3.20	0.003
Associations identified in male fetus pregnancies												
cg00985086	*MCTP1*	0.08 ± 0.02	0.138	0.011 (0.005)	0.19 ± 0.03	0.001	0.011 (0.005)	− 0.01 ± 0.01	0.997	0.012 (0.004)	6.32	1.69 × 10^−8^
cg03215315	*GSTCD; INTS12*	0.07 ± 0.02	0.705	0.012 (0.005)	0.18 ± 0.03	0.009	0.012 (0.005)	− 0.01 ± 0.03	0.998	0.012 (0.005)	4.48	1.61 × 10^−5^
cg23797252	*KNDC1*	0.01 ± 0.003	0.722	0.482 (0.035)	0.02 ± 0.005	0.026	0.480 (0.036)	− 0.01 ± 0.005	0.979	0.484 (0.034)	4.24	3.79 × 10^−5^
cg14065446	*FIBCD1*	0.02 ± 0.01	0.346	0.334 (0.050)	0.04 ± 0.01	0.046	0.336 (0.051)	0.01 ± 0.01	0.991	0.331 (0.048)	2.12	0.035
cg00140191	*FKBP5*	0.10 ± 0.02	0.138	0.032 (0.011)	0.20 ± 0.04	0.046	0.032 (0.012)	− 0.02 ± 0.02	0.990	0.032 (0.011)	4.92	5.07 × 10^−6^
cg16680530	*TATDN1; NDUFB9*	0.08 ± 0.02	0.622	0.012 (0.006)	0.21 ± 0.04	0.046	0.012 (0.006)	− 0.04 ± 0.02	0.953	0.012 (0.006)	5.59	2.98 × 10^−7^
cg24807054	*SFRS18*	0.07 ± 0.01	0.138	0.037 (0.012)	0.15 ± 0.03	0.049	0.036 (0.013)	0.003 ± 0.01	0.999	0.038 (0.011)	4.65	1.24 × 10^−5^
cg18350520	*KIAA0664*	0.01 ± 0.005	0.896	0.894 (0.019)	0.03 ± 0.01	0.049	0.896 (0.014)	− 0.001 ± 0.01	0.999	0.892 (0.023)	2.19	0.033
Associations identified in female fetus pregnancies												
cg04879876	*ZFP36L2; LOC100129726*	0.04 ± 0.04	0.982	0.020 (0.011)	− 0.20 ± 0.06	0.526	0.019 (0.011)	0.28 ± 0.04	7.4 × 10^−7^	0.021(0.011)	− 6.66	3.27 × 10^−10^
cg16661579	*C10orf4*	0.12 ± 0.04	0.686	0.010 (0.006)	− 0.02 ± 0.05	0.998	0.010 (0.006)	0.33 ± 0.06	0.0004	0.010(0.006)	− 4.48	0.00012
cg04777683	*IVNS1ABP*	0.16 ± 0.03	0.110	0.017 (0.009)	0.01 ± 0.05	0.999	0.017 (0.008)	0.31 ± 0.06	0.001	0.017(0.009)	− 3.84	0.00062
cg25928819	*AK055957*	− 0.06 ± 0.02	0.715	0.155 (0.094)	0.01 ± 0.03	0.998	0.147 (0.089)	− 0.14 ± 0.03	0.004	0.163 (0.099)	3.54	0.0012
cg03432641	*SPATS2*	0.08 ± 0.03	0.596	0.011 (0.006)	0.04 ± 0.04	0.977	0.011 (0.006)	0.14 ± 0.03	0.010	0.011 (0.006)	− 2.00	0.048
cg23065793	*LOC100128164; SEC62*	0.07 ± 0.02	0.488	0.021 (0.009)	− 0.01 ± 0.02	0.997	0.021 (0.010)	0.16 ± 0.03	0.010	0.021 (0.009)	− 4.71	6.53 × 10^−5^
cg25861327	*NUSAP1; OIP5*	0.24 ± 0.05	0.146	0.007 (0.005)	0.14 ± 0.09	0.946	0.007 (0.005)	0.35 ± 0.07	0.010	0.007 (0.005)	− 2.21	0.032
cg11149743	*HOXB7*	0.13 ± 0.04	0.496	0.009 (0.011)	0.04 ± 0.05	0.992	0.008 (0.009)	0.22 ± 0.04	0.014	0.010 (0.012)	− 2.81	0.0067
cg10038542	*ENTPD4*	0.05 ± 0.02	0.637	0.026 (0.012)	0.01 ± 0.03	0.997	0.026 (0.012)	0.13 ± 0.03	0.015	0.026 (0.012)	− 2.82	0.0066
cg24737639	*NUP37; C12orf48*	0.09 ± 0.03	0.470	0.016 (0.008)	− 0.04 ± 0.02	0.946	0.017 (0.008)	0.24 ± 0.05	0.019	0.016 (0.007)	− 5.20	1.66 × 10^−5^
cg19714762	*ABHD11*	0.25 ± 0.07	0.445	0.008 (0.007)	0.11 ± 0.12	0.984	0.008 (0.007)	0.42 ± 0.09	0.019	0.009 (0.007)	− 2.07	0.042
cg21490179	*ENTPD3-AS1*	0.01 ± 0.004	0.863	0.056 (0.010)	− 0.01 ± 0.01	0.985	0.055 (0.010)	0.03 ± 0.01	0.019	0.057 (0.011)	− 2.83	0.0066
cg01952989	*MAD1L1*	0.07 ± 0.05	0.971	0.955 (0.097)	− 0.06 ± 0.08	0.992	0.953 (0.111)	0.26 ± 0.05	0.019	0.958(0.080)	− 3.39	0.0018
cg06459916	*KRCC1*	0.08 ± 0.02	0.285	0.009 (0.004)	0.03 ± 0.03	0.981	0.009 (0.004)	0.14 ± 0.03	0.019	0.009 (0.004)	− 2.59	0.012
cg26687565	*MAML3*	0.07 ± 0.02	0.537	0.032 (0.014)	0.004 ± 0.02	0.998	0.031 (0.014)	0.16 ± 0.03	0.019	0.034 (0.015)	− 4.33	0.00018
cg04484842	*MYO9A; SENP8*	0.03 ± 0.01	0.863	0.018 (0.007)	− 0.01 ± 0.02	0.990	0.018 (0.007)	0.08 ± 0.02	0.019	0.018 (0.006)	− 3.18	0.0025
cg25585364	*INSIG2*	0.03 ± 0.01	0.823	0.046 (0.018)	− 0.01 ± 0.02	0.997	0.044 (0.016)	0.09 ± 0.02	0.024	0.048 (0.020)	− 3.54	0.0012
cg22548088	*MLLT1*	0.02 ± 0.01	0.635	0.537 (0.049)	− 0.01 ± 0.01	0.985	0.541 (0.043)	0.04 ± 0.01	0.028	0.533 (0.054)	− 2.24	0.031
cg09062638	*C1QB*	0.06 ± 0.04	0.950	0.937 (0.079)	− 0.11 ± 0.05	0.906	0.939 (0.079)	0.24 ± 0.05	0.029	0.935 (0.080)	− 4.95	1.24 × 10^−6^
cg08130668	*C2orf73*	0.08 ± 0.04	0.888	0.014 (0.008)	− 0.08 ± 0.06	0.970	0.014 (0.008)	0.25 ± 0.05	0.029	0.014 (0.008)	− 4.23	0.00018
cg19715081	*CDK5RAP3*	0.15 ± 0.04	0.503	0.010 (0.006)	− 0.01 ± 0.06	0.999	0.010 (0.007)	0.29 ± 0.06	0.034	0.009 (0.005)	− 3.54	0.0012
cg22830707	*HOXC13*	− 0.02 ± 0.01	0.537	0.172 (0.030)	− 0.01 ± 0.01	0.980	0.173 (0.032)	− 0.03 ± 0.01	0.034	0.171 (0.027)	1.41	0.158
cg11078433	*SLC25A13*	0.01 ± 0.004	0.833	0.040 (0.008)	− 0.01 ± 0.01	0.984	0.038 (0.007)	0.03 ± 0.01	0.036	0.040 (0.008)	− 2.83	0.0066
cg05064665	*PNMT*	0.10 ± 0.04	0.867	0.050 (0.027)	0.01 ± 0.05	0.999	0.047 (0.023)	0.27 ± 0.06	0.036	0.053 (0.030)	− 3.33	0.0020
cg04680746	*NACAD*	0.19 ± 0.05	0.470	0.008 (0.005)	0.07 ± 0.08	0.990	0.008 (0.005)	0.30 ± 0.07	0.036	0.008 (0.005)	− 2.16	0.034
cg13190531	*POLR3B*	0.16 ± 0.06	0.787	0.003 (0.003)	− 0.02 ± 0.09	0.998	0.003 (0.003)	0.39 ± 0.09	0.036	0.004 (0.003)	− 3.22	0.0025
cg24776326	*IRX4*	− 0.02 ± 0.01	0.914	0.597 (0.060)	0.01 ± 0.01	0.977	0.602 (0.055)	− 0.05 ± 0.01	0.036	0.593 (0.065)	4.24	0.00018
cg07576517	*SDCCAG8; CEP170*	0.07 ± 0.02	0.738	0.031 (0.011)	− 0.02 ± 0.04	0.995	0.030 (0.010)	0.16 ± 0.04	0.036	0.033 (0.011)	− 3.18	0.0025
cg23808931	*TMEM183A; TMEM183B*	0.06 ± 0.03	0.820	0.019 (0.014)	− 0.02 ± 0.03	0.990	0.020 (0.014)	0.20 ± 0.05	0.036	0.018 (0.014)	− 3.77	0.00074
cg02376269	*UBR1*	0.06 ± 0.02	0.852	0.036 (0.014)	− 0.01 ± 0.04	0.997	0.034 (0.013)	0.15 ± 0.03	0.036	0.038(0.015)	− 3.20	0.0025
cg10835423	*RAP1A*	0.09 ± 0.03	0.635	0.012 (0.006)	− 0.01 ± 0.04	0.998	0.012 (0.006)	0.19 ± 0.04	0.036	0.012 (0.007)	− 3.53	0.0012
cg03734035	*NDUFB8*	0.11 ± 0.04	0.759	0.011 (0.006)	− 0.03 ± 0.06	0.996	0.011 (0.006)	0.26 ± 0.06	0.042	0.011 (0.007)	− 3.42	0.0017
cg07147063	*TMEM208; LRRC29*	0.03 ± 0.02	0.980	0.022 (0.010)	− 0.04 ± 0.04	0.990	0.021 (0.009)	0.10 ± 0.02	0.042	0.022 (0.010)	− 3.13	0.0030
cg23890800	*FBRSL1*	0.07 ± 0.04	0.947	0.011 (0.007)	− 0.08 ± 0.06	0.972	0.011 (0.007)	0.24 ± 0.05	0.045	0.011 (0.007)	− 4.10	0.00026

^a^Adjusted for maternal age, race/ethnicity, pre-pregnancy BMI, education, job status, gestational age, parity, perceived stress, methylation principal components, genotype principal components, and surrogate variable. Total sample additionally adjusted for fetal sex

^b^All the test statistics are for sex-specific effects except those for cg04879876 and cg09062638 representing opposite effect directions

*CpG* cytosine-(phosphate)-guanine site, *FDR* false discovery rate, *LogFC* logarithm of fold change, *S.E* standard error, *SD* standard deviation

### Correlation between methylation of CpGs and expression of nearby genes in placenta

Higher methylation at cg11364468 (found to be associated with higher social support in the overall sample and male sample) was associated with lower expression of *VGF*. Higher methylation at cg16763895 (found to be associated with higher social support in the overall sample) was associated with lower expression of *ILVBL* (Table 3). *VGF* is a protein-coding gene known to be highly expressed in parts of the brain and neuroendocrine cells (Additional file 1: Figure S5). Several peptide proteins encoded by *VGF* have important roles in brain development and behavioral phenotypes [39] and regulation of energy metabolism [40]. Gene ontologies indicate that the protein encoded by *ILVBL*, which is widely expressed across different tissues (Additional file 1: Figure S6), is involved in fatty acid alpha-oxidation in the endoplasmic reticulum [41] and biosynthesis of isoleucine and valine [42].

**Table 3:** Association between methylation levels of social support-related placental methylation sites and placental expression level of nearby genes (*n* = 75)^a^

CpG	Gene	*β* coeff. ± S.E.	*P*-value	*P*_FDR_
cg16763895	*ILVBL*	− 542.1 ± 151.3	0.0006	0.007
cg16763895	*OR7A17*	− 0.04 ± 0.02	0.0154	0.085
cg11364468	*VGF*	− 0.66 ± 0.22	0.0038	0.037
cg11364468	*MUC17*	− 0.06 ± 0.02	0.0057	0.037

*CpG* cytosine-(phosphate)-guanine site, *S.E* standard error, *FDR* false discovery rate

^a^Only FDR-significant associations are shown

### Functional annotations and regulatory enrichment

CpGs associated with social support in the female sample showed enrichment for DNase 1 hypersensitive sites in fetal brain (*P*_FDR_ < 0.05), but no enrichment was found for the overall or male-specific CpGs associated with social support (Additional file 2: Tables S7–S24). None of the social support-associated CpGs has previously been identified as *cis*-mQTL in placenta [25] which further suggests the observed methylation differences are likely to be the effect of social support rather than that of genetic variants.

### Differentially methylated regions

Analyses of DMRs identified 18, 28, and 22 DMRs associated with social support in the overall, male, and female samples, respectively. Two genes (*KNDC1* and *KIAA0664*) annotating DMRs overlapped with genes annotating CpGs identified in the male sample (Additional file 3: Tables S25–S27).

### Pathway analysis

The genes annotating the top 100 social support-associated CpGs in the overall sample showed enrichment of IPA canonical pathways related to fetal growth, coagulation system, energy metabolism, and neurodevelopment (Table 4). For male-specific CpGs, enrichment was found for pathways related to immune system, cell cycle, tissue growth, and endocrine receptors signaling (Additional file 4: Table S28). For female-specific CpGs, enrichment was found for pathways relevant for immune system, neurodevelopment, and endocrine receptors signaling as well as processes important in placental development and maturation such as cell proliferation and cellular migration (Additional file 4: Table S29).

**Table 4:** Ingenuity pathway analysis canonical pathways of genes annotated to the top 100 social support associated methylation sites in placenta (total sample, *n* = 301)

Ingenuity canonical pathways	Log ***P***-value	Ratio	Molecules
Extrinsic prothrombin activation pathway	2.71	0.125	*F3*, *THBD*
Coagulation system	2.04	0.057	*F3*, *THBD*
White adipose tissue browning pathway	1.72	0.022	*ADCY9*, *FGFR1*, *VGF*
Regulation of eIF4 and p70S6K signaling	1.42	0.017	*AGO3*, *EIF3F*, *ITGAE*
Synaptogenesis signaling pathway	1.38	0.013	*ADCY9*, *ARHGEF7*, *EFNA5*, *THBS2*
FGF signaling	1.33	0.024	*FGFR1*, *FRS2*
Hippo signaling	1.32	0.024	*SCRIB*, *TEAD4*

## Discussion

In this first report of epigenetic signatures of social support in human placentas, we found that the level of prenatal social support during the first trimester of pregnancy is associated with differential methylation of seven CpGs in placenta at delivery. We also identified an additional 42 social support-associated CpGs in placenta dependent on fetal sex. The social support-associated epigenetic signatures in placenta are independent of prenatal stress; hence, social support may have impact on placental methylation even when maternal stress levels are not high. The association between placental expressions of *VGF*, *ILVBL* and *MUC17*, and DNA methylation at two of the social support-associated CpGs hints at the potential gene regulatory roles of the DNA methylation changes. Studies have previously demonstrated the epigenetic regulation of *VGF* [43, 44] and *MUC17* [45, 46] expressions in different tissues. Genes annotated to social support-associated CpGs were enriched for pathways related to the immune system among others. Collectively, our findings support the biological effects of prenatal social support on the in-utero environment which may potentially have fetal programming effects [47], extending previous reports on the relations between social factors during pregnancy and methylation in maternal blood [11] and in placenta of Rhesus monkeys [12].

A positive effect of social support on health and well-being even under low stress environment has long been recognized [2]. While social support may mitigate the negative effects of stress on health outcomes, it is possible that social support independently promotes health and pregnancy outcomes. For example, prenatal social support has been linked to higher newborn leukocyte telomere length [5] and higher birth weight [48–51]—a marker of fetal growth and a predictor of adulthood health outcomes. The enrichment of FGF signaling and Hippo signaling pathways, which are reportedly involved in regulation of telomerase activity [52, 53], also suggests a potential mechanism for the effect of prenatal social support on fetal outcomes.

The enrichment of pathways related to the immune system and cytokines supports shared mechanisms for the potential effects of social support, stress, infections, and other factors. A meta-analytic review has found evidence supporting the link between low social support and inflammation [13]. The quality of social support during pregnancy has also been associated with inflammation during pregnancy and early infancy [14, 15]. Given the link between *MUC17* expression level in different tissues and inflammatory activation [54, 55], our finding of decreased *MUC17* expression with increased methylation at cg11364468 which in turn is associated with higher social support suggests involvement of inflammatory pathways. Therefore, we speculate that prenatal social support may promote fetal outcomes through attenuation of excessive inflammatory activation in placenta in response to various environmental and biological factors. Since psychosocial stress is only one of many proinflammatory environmental factors [56], the positive effect of social support on fetal outcomes may extend beyond pregnancies with high levels of stress.

The placenta has functional roles in fetal neurodevelopment via the “placenta-brain axis,” with potential programming for future mental health outcomes [57]. *VGF* is a protein-coding gene with biased expression in the brain (Figure S5), and its dysregulation has been linked to abnormalities in neural progenitor cell differentiation [58]. In animal studies, dysregulation of *VGF* had effect on brain development and behavioral phenotypes [39], depression-like behaviors [59], and memory consolidation and stress resilience [60, 61]. In humans, *VGF* has been suggested as a biomarker of different neuropsychiatric diseases [62]. The decreased expression of *VGF* associated with hypermethylation of cg11364468, enrichment of CpGs for fetal brain cells, and enrichment of annotated genes for pathways involved in brain development suggest that prenatal social environment may be involved in fetal programming for neuropsychiatric outcomes.

On the other hand, research suggests that *VGF-*derived peptides have an important role in the regulation of energy balance [40]. Although different mechanisms may exist, *VGF* activity in the hypothalamus, which is key in the regulation of feeding and energy metabolism, has been implicated [63, 64]. Increased methylation at cg16763895 associated with decreased expression of *ILVBL* which is involved in oxidation of fatty acids, suggesting fetal programming effect of social support on pathways relevant to energy metabolism. However, further research is needed to elucidate whether the epigenetic changes associated with prenatal social support in placenta are associated with later health outcomes in the offspring.

Our findings indicate sex-specific responses of placental epigenome to prenatal social environment. Nevertheless, pathway analyses revealed convergence in enrichment of canonical pathways such as those related to the immune system for the genes annotated to the top 100 social support associated CpGs in pregnancies with male and female fetuses. Studies have previously demonstrated that epigenetic programming of placenta occurs in a sex-dependent manner [65, 66], and in the case of social support, both converge at immune response and inflammation pathways, despite involvement of different CpG sites. We found hypermethylation of cg00140191 (*FKBP5*) with higher social support in only male pregnancies. Prenatal stress-associated differential methylation of *FKBP5* in placenta has previously been linked to infant neurobehavioral outcomes [67]. Hypomethylation of cg00140191 was reported in peripheral blood of adolescents who had childhood victimization [68]. Overall, our findings indicate that sex-specific analyses offer the opportunity for better understanding the effects of social support and perhaps other environmental factors on placental epigenome. The potential implications of these sex differences on long term health outcomes may be crucial for understanding health disparities in men and women.

We acknowledge the following limitations arising from our design. First, our study may have been underpowered to detect additional associations because of relatively small sample size, particularly for subgroup and gene expression analyses. However, the post hoc power estimates indicate that most of the DNA methylation effect sizes were adequately powered. Second, the participants were selected to study low risk pregnancy, and this may have led to exclusion of individuals with low social support, e.g., individuals with drug addiction or psychiatric disorders. Finally, the level of social support may have changed later during pregnancy. Despite these limitations, we found novel CpGs in placenta associated with social support which withstood correction for multiple testing and adjustment for several important confounders, including estimates of placental cell composition and genetic ancestry. Our data support placental epigenetic programming effect of social support in racially diverse pregnant women with implications for offspring neuropsychiatric and cardiometabolic health. These findings need to be interpreted in the light of the shared genetic risk between loneliness, neuropsychiatric and cardiovascular morbidities [69].

## Conclusions

We identified placental DNA methylation changes associated with prenatal social support independent of the level of prenatal stress during pregnancy. Some of these placental DNA methylation changes varied by fetal sex. The genes annotated to the DNA methylation loci are enriched for pathways involved in the immune system, placental growth and maturation, brain development, and energy metabolism. Research in molecular mechanisms of effect of social support on health outcomes may provide useful insight for developing interventions that promote fetal neurodevelopment. Further research is needed to replicate the findings and identify molecular mechanisms of effect of the broader social environment on pregnancy and fetal outcomes.

## Supplementary Information

**Additional file 1: Fig S1.** Manhattan plot and QQ plot of CpGs in placenta associated with maternal social support for all pregnancies. **Fig S2.** Manhattan plot and QQ plot of CpGs in placenta associated with maternal social support for pregnancies with male fetus. **Fig S3.** Manhattan plot and QQ plot of CpGs in placenta associated with maternal social support for pregnancies with female fetus. **Fig S4.** Distribution of post hoc power analyzed for DNA methylation effect sizes. **Fig S5.** Tissue expression of *VGF.* **Fig S6.** Tissue expression of *ILVBL.* **Table S1.** Characteristics of study participants who provided placenta samples and those who did not. **Table S2.** Sensitivity analysis for CpGs in placenta associated with maternal social support. **Table S3.** CpGs in placenta associated with maternal social support in pregnancies with male fetus. **Table S4.** CpGs in placenta associated with maternal social support in pregnancies with female fetus. **Table S5.** Sensitivity analysis for CpGs in placenta associated with maternal social support in pregnancies with male fetus. **Table S6.** Sensitivity analysis for CpGs in placenta associated with maternal social support in pregnancies with female fetus.

**Additional file 2: Tables S7–S12.** Enrichment and depletion of CpGs in placenta associated with maternal social support in different cell types and tissues for DNAase hypersensitive sites, chromatin marks and H3 histone marks in all pregnancies. Tables **S13–S18.** Enrichment and depletion of CpGs in placenta associated with maternal social support in different cell types and tissues for DNAase hypersensitive sites, chromatin marks and H3 histone marks in pregnancies with male fetus. **Tables S19–S24.** Enrichment and depletion of CpGs in placenta associated with maternal social support in different cell types and tissues for DNAase hypersensitive sites, chromatin marks and H3 histone marks in pregnancies with female fetus.

**Additional file 3: Table S25.** Differentially methylated regions in placenta associated with maternal social support in all pregnancies. **Table S26.** Differentially methylated regions in placenta associated with maternal social support in pregnancies with male fetus. **Table S27.** Differentially methylated regions in placenta associated with maternal social support in pregnancies with female fetus.

**Additional file 4: Table S28.** Ingenuity canonical pathways of genes annotated to the top 100 CpGs in placenta associated with maternal social support in pregnancies with male fetus. **Table S29.** Ingenuity canonical pathways of genes annotated to the top 100 CpGs in placenta associated with maternal social support in pregnancies with female fetus.

## Data Availability

The placental DNA methylation, genotype, and gene expression data are available through dbGaP with accession number phs001717.v1.p1 [25, 70]. The analytic codes for the current study are available upon request to the corresponding author.

## Abbreviations

BMIQ: Beta Mixture Quantile dilation
CED: Concordant effect direction
CpG: Cytosine-phosphate-guanine site
DMR: Differentially methylated region
ESSI: ENRICHD Social Support Instrument
EWAS: Epigenome-wide association study
FDR: False discovery rate
FGS: Fetal growth study
GTEx: Genotype-Tissue Expression Project
IPA: Ingenuity pathway analysis
mQTL: Methylation quantitative trait locus
NICHD: National Institute of Child Health and Human Development
OED: Opposite effect direction
PSS-10: Perceived Stress Scale 10
QQ: Quantile-quantile
SNP: Single nucleotide polymorphism
SSE: Single sex effect
SVA: Surrogate variable analysis

## References

[1] Thoits PA (2011). Mechanisms linking social ties and support to physical and mental health. J Health Soc Behav.

[2] Cohen S, Wills TA (1985). Stress, social support, and the buffering hypothesis. Psychol Bull.

[3] Field RJ, Schuldberg D (2011). Social-support moderated stress: a nonlinear dynamical model and the stress-buffering hypothesis. Nonlinear Dynamics Psychol Life Sci.

[4] Hornstein EA, Eisenberger NI (2017). Unpacking the buffering effect of social support figures: social support attenuates fear acquisition. PLoS One.

[5] Verner G, Epel E, Lahti-Pulkkinen M, Kajantie E, Buss C, Lin J (2021). Maternal psychological resilience during pregnancy and newborn telomere length: a prospective study. Am J Psychiatry.

[6] Katzow M, Messito MJ, Mendelsohn AL, Scott MA, Gross RS (2019). The Protective effect of prenatal social support on infant adiposity in the first 18 months of life. J Pediatr.

[7] Uchino BN (2009). Understanding the links between social support and physical health: a life-span perspective with emphasis on the separability of perceived and received support. Perspect Psychol Sci.

[8] Rondinone O, Murgia A, Costanza J, Tabano S, Camanni M, Corsaro L, et al. Extensive placental methylation profiling in normal pregnancies. Int J Mol Sci. 2021;22(4):2136. 10.3390/ijms22042136 PMC7924820 33669975

[9] Novakovic B, Yuen RK, Gordon L, Penaherrera MS, Sharkey A, Moffett A (2011). Evidence for widespread changes in promoter methylation profile in human placenta in response to increasing gestational age and environmental/stochastic factors. BMC Genomics.

[10] Ryan J, Mansell T, Fransquet P, Saffery R (2017). Does maternal mental well-being in pregnancy impact the early human epigenome?. Epigenomics..

[11] Surkan PJ, Hong X, Zhang B, Nawa N, Ji H, Tang WY (2020). Can social support during pregnancy affect maternal DNA methylation? Findings from a cohort of African-Americans. Pediatr Res.

[12] Massart R, Suderman MJ, Nemoda Z, Sutti S, Ruggiero AM, Dettmer AM (2017). The signature of maternal social rank in placenta deoxyribonucleic acid methylation profiles in rhesus monkeys. Child Dev.

[13] Uchino BN, Trettevik R, Kent de Grey RG, Cronan S, Hogan J, Baucom BRW (2018). Social support, social integration, and inflammatory cytokines: a meta-analysis. Health Psychol.

[14] Ross KM, Miller G, Qadir S, Keenan-Devlin L, Leigh AKK, Borders A (2017). Close relationship qualities and maternal peripheral inflammation during pregnancy. Psychoneuroendocrinology..

[15] Ross KM, Thomas JC, Letourneau NL, Campbell TS, Giesbrecht GF (2019). Partner social support during pregnancy and the postpartum period and inflammation in 3-month-old infants. Biol Psychol.

[16] Grewal J, Grantz KL, Zhang C, Sciscione A, Wing DA, Grobman WA (2018). Cohort Profile: NICHD fetal growth studies-singletons and twins. Int J Epidemiol.

[17] Inkster AM, Yuan V, Konwar C, Matthews AM, Brown CJ, Robinson WP (2021). A cross-cohort analysis of autosomal DNA methylation sex differences in the term placenta. Biol Sex Differ.

[18] Chatterjee S, Zeng X, Ouidir M, Tesfaye M, Zhang C, Tekola-Ayele F (2022). Sex-specific placental gene expression signatures of small for gestational age at birth. Placenta..

[19] Andrews SV, Yang IJ, Froehlich K, Oskotsky T, Sirota M (2022). Large-scale placenta DNA methylation integrated analysis reveals fetal sex-specific differentially methylated CpG sites and regions. Sci Rep.

[20] Tekola-Ayele F, Workalemahu T, Gorfu G, Shrestha D, Tycko B, Wapner R (2019). Sex differences in the associations of placental epigenetic aging with fetal growth. Aging (Albany NY).

[21] Tesfaye M, Chatterjee S, Zeng X, Joseph P, Tekola-Ayele F (2021). Impact of depression and stress on placental DNA methylation in ethnically diverse pregnant women. Epigenomics..

[22] Xu R, Hong X, Zhang B, Huang W, Hou W, Wang G (2021). DNA methylation mediates the effect of maternal smoking on offspring birthweight: a birth cohort study of multi-ethnic US mother-newborn pairs. Clin Epigenetics.

[23] Vaglio J, Conard M, Poston WS, O'Keefe J, Haddock CK, House J (2004). Testing the performance of the ENRICHD Social Support Instrument in cardiac patients. Health Qual Life Outcomes.

[24] Cohen S, Kamarck T, Mermelstein R (1983). A global measure of perceived stress. J Health Soc Behav.

[25] Delahaye F, Do C, Kong Y, Ashkar R, Salas M, Tycko B (2018). Genetic variants influence on the placenta regulatory landscape. PLoS Genet.

[26] Shrestha D, Ouidir M, Workalemahu T, Zeng X, Tekola-Ayele F (2020). Placental DNA methylation changes associated with maternal prepregnancy BMI and gestational weight gain. Int J Obes.

[27] Teschendorff AE, Marabita F, Lechner M, Bartlett T, Tegner J, Gomez-Cabrero D (2013). A beta-mixture quantile normalization method for correcting probe design bias in Illumina Infinium 450 k DNA methylation data. Bioinformatics..

[28] Du P, Zhang X, Huang CC, Jafari N, Kibbe WA, Hou L (2010). Comparison of Beta-value and M-value methods for quantifying methylation levels by microarray analysis. BMC Bioinformatics.

[29] Leek JT, Johnson WE, Parker HS, Jaffe AE, Storey JD (2012). The sva package for removing batch effects and other unwanted variation in high-throughput experiments. Bioinformatics..

[30] Yuan V, Hui D, Yin Y, Penaherrera MS, Beristain AG, Robinson WP (2021). Cell-specific characterization of the placental methylome. BMC Genomics.

[31] van Iterson M, van Zwet EW, Heijmans BT, Consortium B (2017). Controlling bias and inflation in epigenome- and transcriptome-wide association studies using the empirical null distribution. Genome Biol.

[32] Randall JC, Winkler TW, Kutalik Z, Berndt SI, Jackson AU, Monda KL (2013). Sex-stratified genome-wide association studies including 270,000 individuals show sexual dimorphism in genetic loci for anthropometric traits. PLoS Genet.

[33] Matthew Suderman JRS, French R, Arathimos R, Simpkin A, Tilling K (2018). dmrff: identifying differentially methylated regions efficiently with power and control.

[34] Breeze CE, Paul DS, van Dongen J, Butcher LM, Ambrose JC, Barrett JE (2016). eFORGE: a tool for identifying cell type-specific signal in epigenomic data. Cell Rep.

[35] Tekola-Ayele F, Zeng X, Ouidir M, Workalemahu T, Zhang C, Delahaye F (2020). DNA methylation loci in placenta associated with birthweight and expression of genes relevant for early development and adult diseases. Clin Epigenetics.

[36] Mitchell PH, Powell L, Blumenthal J, Norten J, Ironson G, Pitula CR (2003). A short social support measure for patients recovering from myocardial infarction: the ENRICHD Social Support Inventory. J Cardpulm Rehabil.

[37] Grobman WA, Parker C, Wadhwa PD, Willinger M, Simhan H, Silver B (2016). Racial/ethnic disparities in measures of self-reported psychosocial states and traits during pregnancy. Am J Perinatol.

[38] Nordin M, Nordin S (2013). Psychometric evaluation and normative data of the Swedish version of the 10-item perceived stress scale. Scand J Psychol.

[39] Mizoguchi T, Minakuchi H, Ishisaka M, Tsuruma K, Shimazawa M, Hara H (2017). Behavioral abnormalities with disruption of brain structure in mice overexpressing VGF. Sci Rep.

[40] Lewis JE, Brameld JM, Jethwa PH (2015). Neuroendocrine role for VGF. Front Endocrinol (Lausanne).

[41] Kitamura T, Seki N, Kihara A (2017). Phytosphingosine degradation pathway includes fatty acid alpha-oxidation reactions in the endoplasmic reticulum. Proc Natl Acad Sci U S A.

[42] Gaudet P, Livstone MS, Lewis SE, Thomas PD (2011). Phylogenetic-based propagation of functional annotations within the Gene Ontology consortium. Brief Bioinform.

[43] Brait M, Maldonado L, Noordhuis MG, Begum S, Loyo M, Poeta ML (2013). Association of promoter methylation of VGF and PGP9.5 with ovarian cancer progression. PLoS One.

[44] Marwitz S, Heinbockel L, Scheufele S, Nitschkowski D, Kugler C, Perner S (2017). Epigenetic modifications of the VGF gene in human non-small cell lung cancer tissues pave the way towards enhanced expression. Clin Epigenetics.

[45] Jiang Z, Wang H, Li L, Hou Z, Liu W, Zhou T (2019). Analysis of TGCA data reveals genetic and epigenetic changes and biological function of MUC family genes in colorectal cancer. Future Oncol.

[46] Lin S, Zhang Y, Hu Y, Yang B, Cui J, Huang J (2019). Epigenetic downregulation of MUC17 by H. pylori infection facilitates NF-kappaB-mediated expression of CEACAM1-3S in human gastric cancer. Gastric Cancer.

[47] Shallie PD, Naicker T (2019). The placenta as a window to the brain: a review on the role of placental markers in prenatal programming of neurodevelopment. Int J Dev Neurosci.

[48] Nkansah-Amankra S, Dhawain A, Hussey JR, Luchok KJ (2010). Maternal social support and neighborhood income inequality as predictors of low birth weight and preterm birth outcome disparities: analysis of South Carolina Pregnancy Risk Assessment and Monitoring System survey, 2000-2003. Matern Child Health J.

[49] Lee HY, Oh J, Perkins JM, Heo J, Subramanian SV (2019). Associations between maternal social capital and infant birth weight in three developing countries: a cross-sectional multilevel analysis of Young Lives data. BMJ Open.

[50] Paredes Mondragon CV, Molano Dorado H, Martinez Gomez SY, Ortiz Martinez RA, Arias Linthon S, Lopez Benavides AC (2019). Relationship between the absence of adequate social support during pregnancy and low birth weight. Rev Colomb Psiquiatr (Engl Ed).

[51] Wado YD, Afework MF, Hindin MJ (2014). Effects of maternal pregnancy intention, depressive symptoms and social support on risk of low birth weight: a prospective study from southwestern Ethiopia. PLoS One.

[52] Zou Y, Tong HJ, Li M, Tan KS, Cao T (2017). Telomere length is regulated by FGF-2 in human embryonic stem cells and affects the life span of its differentiated progenies. Biogerontology..

[53] Zhang Q, Liu N, Bai J, Zhou Q, Mao J, Xu L (2020). Human telomerase reverse transcriptase is a novel target of Hippo-YAP pathway. FASEB J.

[54] Hammoudeh SM, Hammoudeh AM, Bhamidimarri PM, Al Safar H, Mahboub B, Kunstner A (2021). Systems immunology analysis reveals the contribution of pulmonary and extrapulmonary tissues to the immunopathogenesis of severe COVID-19 patients. Front Immunol.

[55] Schneider H, Berger E, Dolan B, Martinez-Abad B, Arike L, Pelaseyed T (2019). The human transmembrane mucin MUC17 responds to TNFalpha by increased presentation at the plasma membrane. Biochem J.

[56] Rohleder N (2014). Stimulation of systemic low-grade inflammation by psychosocial stress. Psychosom Med.

[57] Rosenfeld CS (2021). The placenta-brain-axis. J Neurosci Res.

[58] Hwang I, Pan H, Yao J, Elemento O, Zheng H, Paik J. CIC is a critical regulator of neuronal differentiation. JCI. Insight. 2020;5(9):e135826. 10.1172/jci.insight.135826. 10.1172/jci.insight.135826 PMC7253013 32229723

[59] Jiang C, Lin WJ, Sadahiro M, Labonte B, Menard C, Pfau ML (2018). VGF function in depression and antidepressant efficacy. Mol Psychiatry.

[60] Lin WJ, Jiang C, Sadahiro M, Bozdagi O, Vulchanova L, Alberini CM (2015). VGF and its C-terminal peptide TLQP-62 regulate memory formation in hippocampus via a BDNF-TrkB-dependent mechanism. J Neurosci.

[61] Lin WJ, Zhao Y, Li Z, Zheng S, Zou JL, Warren NA (2021). An increase in VGF expression through a rapid, transcription-independent, autofeedback mechanism improves cognitive function. Transl Psychiatry.

[62] Quinn JP, Kandigian SE, Trombetta BA, Arnold SE, Carlyle BC (2021). VGF as a biomarker and therapeutic target in neurodegenerative and psychiatric diseases. Brain Commun.

[63] Foglesong GD, Huang W, Liu X, Slater AM, Siu J, Yildiz V (2016). Role of hypothalamic VGF in energy balance and metabolic adaption to environmental enrichment in mice. Endocrinology..

[64] Lewis JE, Brameld JM, Hill P, Cocco C, Noli B, Ferri GL (2017). Hypothalamic over-expression of VGF in the Siberian hamster increases energy expenditure and reduces body weight gain. PLoS One.

[65] Dunn GA, Morgan CP, Bale TL (2011). Sex-specificity in transgenerational epigenetic programming. Horm Behav.

[66] Gabory A, Attig L, Junien C (2009). Sexual dimorphism in environmental epigenetic programming. Mol Cell Endocrinol.

[67] Monk C, Feng T, Lee S, Krupska I, Champagne FA, Tycko B (2016). Distress during pregnancy: epigenetic regulation of placenta glucocorticoid-related genes and fetal neurobehavior. Am J Psychiatry.

[68] Marzi SJ, Sugden K, Arseneault L, Belsky DW, Burrage J, Corcoran DL (2018). Analysis of DNA methylation in young people: limited evidence for an association between victimization stress and epigenetic variation in blood. Am J Psychiatry.

[69] Rodevand L, Bahrami S, Frei O, Lin A, Gani O, Shadrin A (2021). Polygenic overlap and shared genetic loci between loneliness, severe mental disorders, and cardiovascular disease risk factors suggest shared molecular mechanisms. Transl Psychiatry.

[70] NCBI. Genetic variants influence on the placenta regulatory landscape. NCBI: database of Genotypes and Phenotypes. https://www.ncbi.nlm.nih.gov/projects/gap/cgi-bin/study.cgi?study_id=phs001717.v1.p1 (2019).

