# Supplementary material for: Prenatal social support in low-risk pregnancy shapes placental epigenome
Source: BMC Med. 2023 Jan 8;21:12. doi: 10.1186/s12916-022-02701-w (PMC9827682; doi:10.1186/s12916-022-02701-w)
Supplement: Supplementary file 1 — Additional file 1: Fig S1. Manhattan plot and QQ plot of CpGs in placenta associated with maternal social support for all pregnancies. Fig S2. Manhattan plot and QQ plot of CpGs in placenta associated with maternal social support for pregnancies with male fetus. Fig S3. Manhattan plot and QQ plot of CpGs in placenta associated with maternal social support for pregnancies with female fetus. Fig S4. Distribution of post hoc power analyzed for DNA methylation effect sizes. Fig S5. Tissue expression of VGF. Fig S6. Tissue expression of ILVBL. Table S1. Characteristics of study participants who provided placenta samples and those who did not. Table S2. Sensitivity analysis for CpGs in placenta associated with maternal social support. Table S3. CpGs in placenta associated with maternal social support in pregnancies with male fetus. Table S4. CpGs in placenta associated with maternal social support in pregnancies with female fetus. Table S5. Sensitivity analysis for CpGs in placenta associated with maternal social support in pregnancies with male fetus. Table S6. Sensitivity analysis for CpGs in placenta associated with maternal social support in pregnancies with female fetus. [file 12916_2022_2701_MOESM1_ESM.docx]

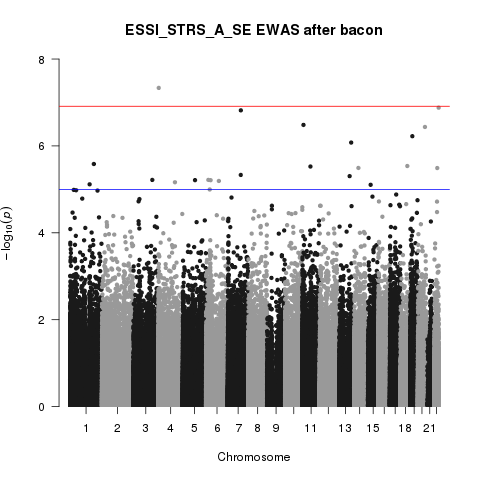


**Figure S1:** Manhattan plot (left), and Q-Q plot (right) of CpGs in placenta and their association with maternal social support during pregnancy for the total sample.


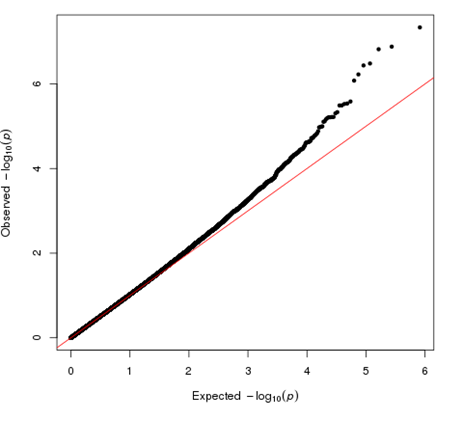


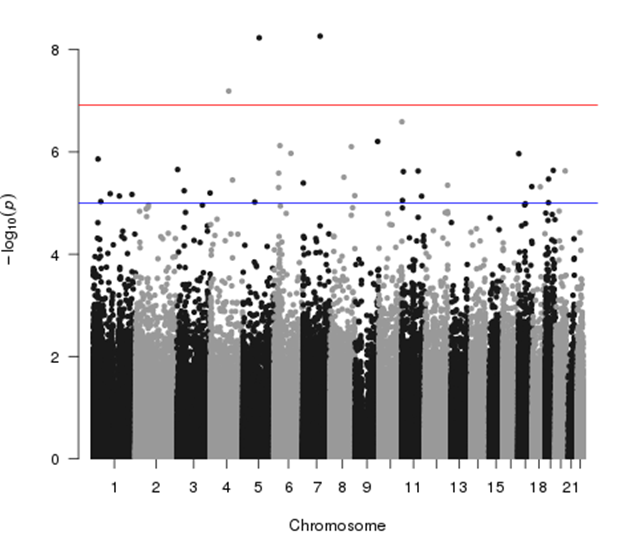


**Figure S2**: Manhattan plot (left), and Q-Q plot (right) of CpGs in placenta and their association with maternal social support during pregnancies with male fetus.


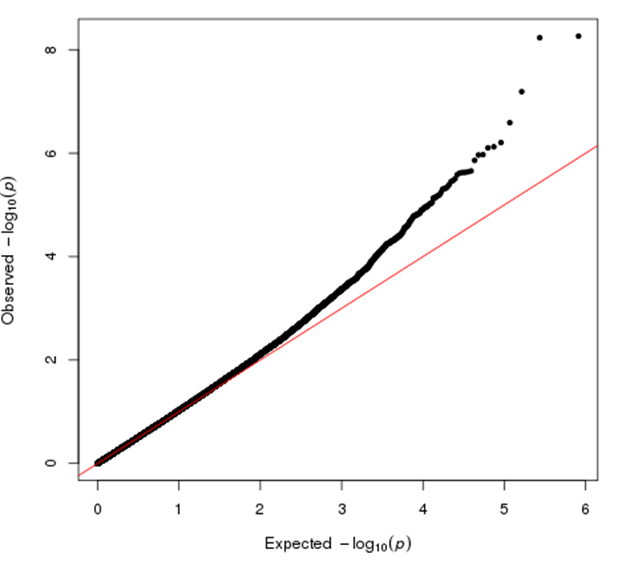


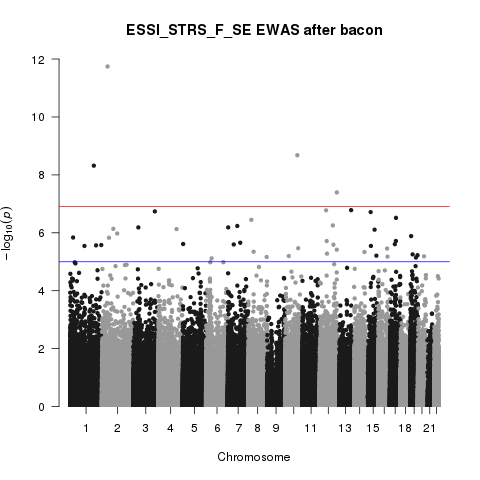


**Figure S3.** Manhattan plot (left), and Q-Q plot (right) of CpGs in placenta and their association with maternal social support during pregnancies with female fetus.


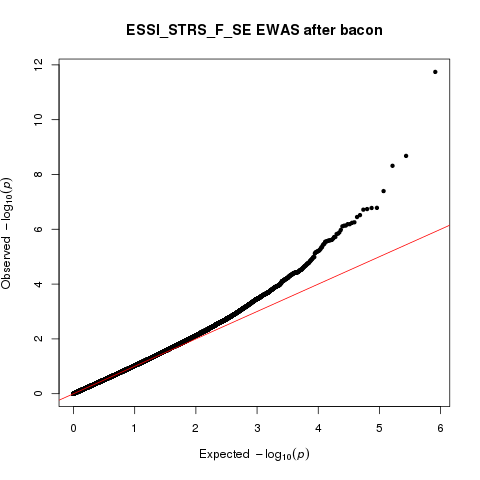


**Figure S4:** The distribution of *post hoc* power analyzed for DNA methylation effect sizes using two tailed tests with probability of error (α=0.05) and sample sizes of 301 and 150.


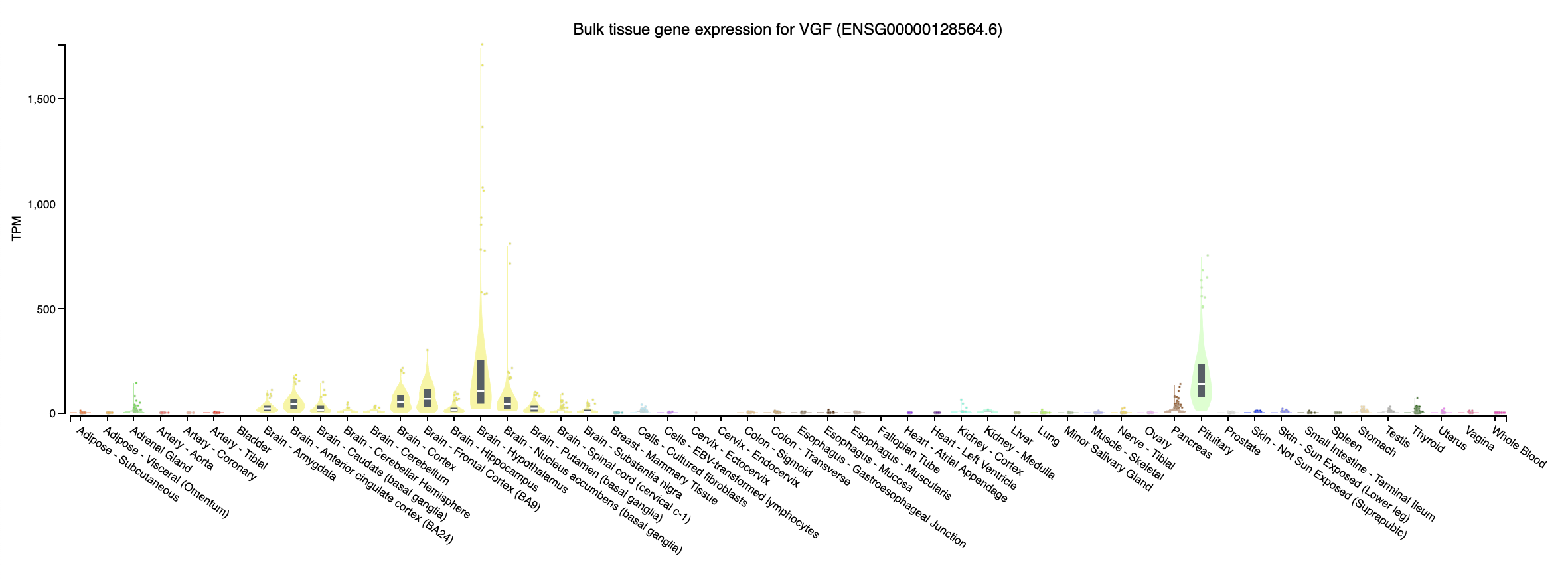


**Figure S5:** Tissue expression of *VGF* (nerve growth factor inducible) (adapted from GTEX portal)


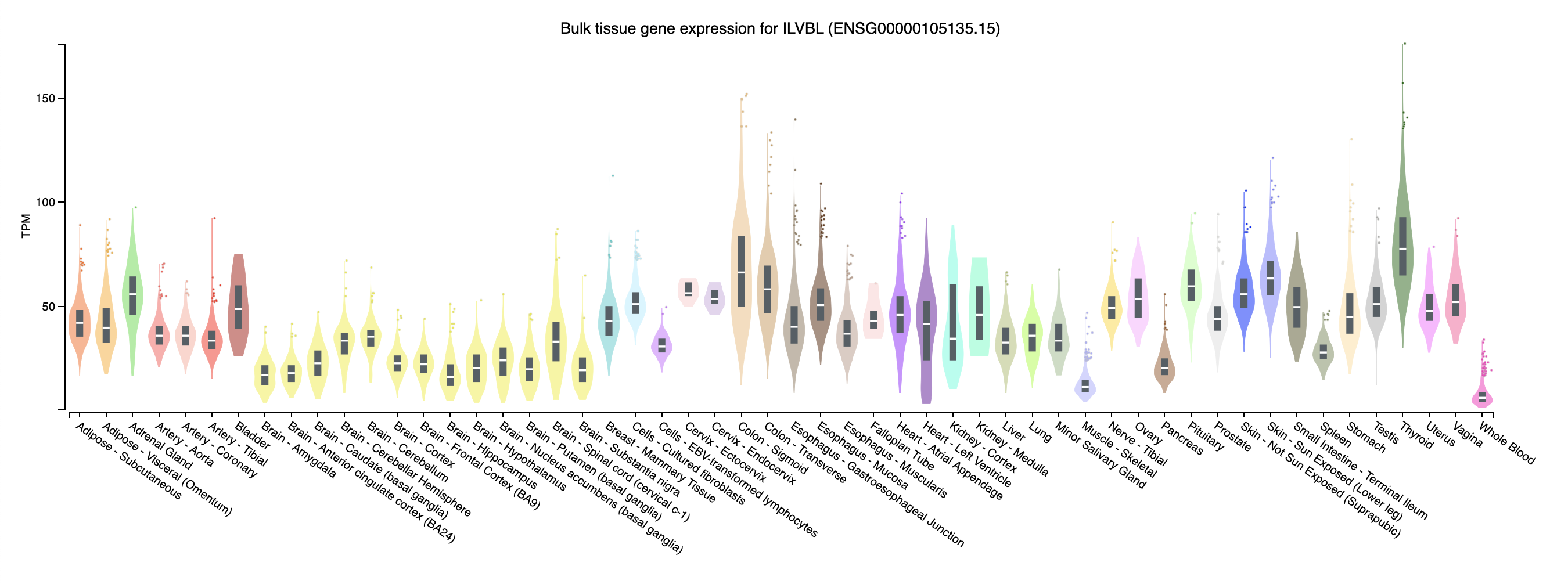


**Figure S6:** Tissue expression of *ILVBL* (IlvB Acetolactate Synthase Like) (adapted from GTEX portal)

| **Table S1: Characteristics of participants of the Fetal Growth study who provided placenta samples compared to those who did not.** | | | |
| --- | --- | --- | --- |
| NICHD Fetal Growth Study | Women who provided placenta (n=301) | Women who did not provide  placenta (n=2501) | P-value |
|  | Mean (SD)/ n (%) | Mean (SD)/ n (%) |  |
| Race/ethnicity |  |  | 0.15 |
| Non-Hispanic White | 77 (25.6%) | 674 (26.9%) |  |
| Non-Hispanic Black | 72 (23.9%) | 709 (28.3%) |  |
| Hispanic | 102 (34%) | 701 (28.0%) |  |
| Asian | 50 (16.7%) | 417 (16.7%) |  |
| Maternal age (years) | 27.7 (5.3) | 28.2 (5.5) | 0.10 |
| Infant sex |  |  | 0.81 |
| Female | 149 (49.5%) | 1157 (51.3%) |  |
| Male | 152 (50.5%) | 1100 (48.7%) |  |
| Gestational age (weeks) | 39.5 (1.1) | 39.1 (2.0) | 6.67E-6 |
| Parity |  |  | 0.85 |
| Parous | 155 (53.3%) | 1179 (47%) |  |
| Nulliparous | 136 (46.7%) | 1132 (53%) |  |
| Educational status |  |  | 0.92 |
| High school and above | 264 (87.7%) | 2210 (88%) |  |
| Below high school | 37 (12.3%) | 289 (12%) |  |
| Marital status |  |  | 0.73 |
| Married | 226 (75%) | 1856 (74%) |  |
| Others | 74 (25%) | 642 (26%) |  |
| Job status |  |  | 1.00 |
| Employed/student | 211 (70%) | 1751 (70%) |  |
| Unemployed? | 89 (30%) | 747 (30%) |  |
| ESSI score | 22.63 (3.25) | 22.35 (3.49) | 0.17 |
| Stress score | 10.93 (5.91) | 11.64 (6.32) | 0.07 |

| **Table S2: Sensitivity analysis for CpGs in placenta associated with level of social support during pregnancy (n=301)** | | | | | | | | | | |
| --- | --- | --- | --- | --- | --- | --- | --- | --- | --- | --- |
| CpG | Gene | Chr: Position | Relation to Gene | Relation to Island | Methylation LogFC (S.E)^1^ | P-value^1^ | P_FDR_^1^ | Methylation LogFC (S.E)^2^ | P- value^2^ | P_FDR_^2^ |
| cg14806252 | *HAUS3* | 4:2244001 | TSS200 | Island | 0.22 (0.04) | 1.6×10^-8^ | 1.1×10^-7^ | 0.22 (0.04) | 1.4×10^-8^ | 4.9×10^-8^ |
| cg01924481 | *SBF1* | 22:50898563 | Body | Island | 0.01 (0.003) | 7.5×10^-6^ | 7.5×10^-6^ | 0.02 (0.003) | 4.3×10^-7^ | 5.0×10^-7^ |
| cg11364468 | *VGF* | 7:100807505 | Body | Island | 0.10 (0.02) | 7.1×10^-7^ | 1.7×10^-6^ | 0.11 (0.02) | 2.5×10^-8^ | 5.8×10^-8^ |
| cg00549575 | *EIF3F* | 11:8008752 | TSS200 | N_Shore | 0.07 (0.02) | 3.1×10^-6^ | 4.3×10^-6^ | 0.08 (0.02) | 2.2×10^-7^ | 3.8×10^-7^ |
| cg19499754 | *FAM210B* | 20:54919155 |  | Island | 0.11 (0.02) | 2.7×10^-7^ | 9.4×10^-7^ | 0.12 (0.02) | 2.8×10^-9^ | 1.9×10^-8^ |
| cg16763895 | *ILVBL* | 19:15235973 | 5'UTR | Island | 0.07 (0.01) | 4.9×10^-6^ | 5.7×10^-6^ | 0.07 (0.01) | 2.7×10^-7^ | 3.8×10^-7^ |
| cg02672368 | *ARHGEF7* | 13:111805930 | Body; TSS200 | Island | 0.12 (0.03) | 1.9×10^-6^ | 3.3×10^-6^ | 0.12 (0.03) | 5.6×10^-6^ | 5.6×10^-6^ |
| ^1^ Model is adjusted for maternal age, race/ethnicity, pre-pregnancy BMI, education, job status, gestational age, parity, fetal sex, perceived stress, methylation PCs, genotype PCs, surrogate variable, and estimated cell composition.  ^2^ Model is adjusted for fetal sex, perceived stress, methylation PCs, genotype PCs, surrogate variable.  FDR – false discovery rate; LogFC – logarithm of fold change; S.E – standard error | | | | | | | | | | |

| **Table S3: CpGs in placenta associated with level of social support during pregnancy with male fetus FGS data (n=152)** | | | | | | | |
| --- | --- | --- | --- | --- | --- | --- | --- |
| CpG | Gene | Chr: Position | Relation to Gene | Relation to Island | Methylation LogFC (S.E.) | P-value | P_FDR_ |
| cg11364468 | *VGF* | 7:100807505 | Body | Island | 0.21 (0.04) | 5.5×10^-9^ | 0.001 |
| cg00985086 | *MCTP1* | 5:94620134 | 1stExon | Island | 0.19 (0.03) | 5.9×10^-9^ | 0.001 |
| cg03215315 | *GSTCD; [INTS12]* | 4:106629983 | 1stExon; 5'UTR; [TSS200] | Island | 0.18 (0.03) | 6.5×10^-8^ | 0.009 |
| cg23797252 | *KNDC1* | 10:135014804 | Body | Island | 0.02 (0.005) | 2.6×10^-7^ | 0.026 |
| cg14065446 | *FIBCD1* | 9:133818825 |  | S_Shelf | 0.04 (0.01) | 6.3×10^-7^ | 0.046 |
| cg00140191 | *FKBP5* | 6:35656242 | 5'UTR | Island | 0.20 (0.04) | 7.6×10^-7^ | 0.046 |
| cg16680530 | *TATDN1; [NDUFB9]* | 8:125551329 | TSS200; [TSS200] | Island | 0.21 (0.04) | 7.9×10^-7^ | 0.046 |
| cg24807054 | *SFRS18* | 6:99873402 | TSS200 | S_Shore | 0.15 (0.03) | 1.1×10^-6^ | 0.049 |
| cg18350520 | *KIAA0664* | 17:2595812 | Body | Island | 0.03 (0.01) | 1.1×10^-6^ | 0.049 |
| Model is adjusted for maternal age, race/ethnicity, pre-pregnancy BMI, education, job status, gestational age, parity, perceived stress, methylation PCs, genotype PCs, and surrogate variable.  FDR – false discovery rate; LogFC – logarithm of fold change; S.E. – standard error | | | | | | | |

| **Table S4: CpGs in placenta associated with level of social support during pregnancy with female fetus FGS data (n=149)** | | | | | | | | |
| --- | --- | --- | --- | --- | --- | --- | --- | --- |
| CpG | Gene | Chr: Position | Relation to Gene | Relation to Island | Methylation LogFC (S.E.) | P-value | P_FDR_ |  |
| cg04879876 | *ZFP36L2; [LOC100129726]* | 2:43453558 | 1stExon; 5'UTR; [TSS1500] | Island | 0.28 (0.04) | 1.8×10^-12^ | 7.4×10^-7^ |  |
| cg16661579 | *C10orf4* | 10:95462206 | 5'UTR;1stExon | Island | 0.33 (0.06) | 2.1×10^-9^ | 0.0004 |  |
| cg04777683 | *IVNS1ABP* | 1:185286462 | TSS200 | Island | 0.31 (0.06) | 4.8×10^-9^ | 0.0007 |  |
| cg25928819 | *AK055957* | 12:133484026 |  | N_Shore | -0.14 (0.03) | 4.0×10^-8^ | 0.0041 |  |
| cg02672368 | *ARHGEF7* | 13:111805930 | Body; TSS200 | Island | 0.21 (0.04) | 1.7×10^-7^ | 0.0099 |  |
| cg03432641 | *SPATS2* | 12:49760606 | TSS200 | Island | 0.14 (0.03) | 1.7×10^-7^ | 0.0099 |  |
| cg23065793 | *LOC100128164; [SEC62]* | 3:169684395 | Body; [TSS200] | Island | 0.16 (0.03) | 1.8×10^-7^ | 0.0099 |  |
| cg25861327 | *NUSAP1; [OIP5]* | 15:41625136 | 1stExon; 5'UTR; [TSS1500] | Island | 0.35 (0.07) | 1.9×10^-7^ | 0.0099 |  |
| cg11149743 | *HOXB7* | 17:46688419 | TSS200 | Island | 0.22 (0.04) | 3.1×10^-7^ | 0.0139 |  |
| cg10038542 | *ENTPD4* | 8:23315318 | TSS200 | Island | 0.13 (0.03) | 3.6×10^-7^ | 0.0146 |  |
| cg24737639 | *NUP37; [C12orf48]* | 12:102513781 | TSS1500; [TSS200] | Island | 0.24 (0.05) | 5.6×10^-7^ | 0.0188 |  |
| cg19714762 | *ABHD11* | 7:73153162 | 1stExon; Body; 5'UTR | Island | 0.42 (0.09) | 5.8×10^-7^ | 0.0188 |  |
| cg21490179 | *ENTPD3-AS1* | 3:40494492 |  | Island | 0.03 (0.01) | 6.5×10^-7^ | 0.0188 |  |
| cg01952989 | *MAD1L1* | 7:1986334 | Body | OpenSea | 0.26 (0.05) | 6.5×10^-7^ | 0.0188 |  |
| cg06459916 | *KRCC1* | 2:88355002 | 5'UTR | Island | 0.14 (0.03) | 7.3×10^-7^ | 0.0188 |  |
| cg26687565 | *MAML3* | 4:141072046 | Body | Island | 0.16 (0.03) | 7.4×10^-7^ | 0.0188 |  |
| cg04484842 | *MYO9A; [SENP8]* | 15:72410733 | TSS1500; [1stExon; 5'UTR] | Island | 0.08 (0.02) | 7.8×10^-7^ | 0.0188 |  |
| cg25585364 | *INSIG2* | 2:118846169 | 5'UTR | Island | 0.09 (0.02) | 1.1×10^-6^ | 0.0241 |  |
| cg22548088 | *MLLT1* | 19:6271786 | Body | N_Shore | 0.04 (0.01) | 1.3×10^-6^ | 0.0281 |  |
| cg09062638 | *C1QB* | 1:23003490 |  | Island | 0.24 (0.05) | 1.5×10^-6^ | 0.0289 |  |
| cg08130668 | *C2orf73* | 2:54557891 | TSS200 | Island | 0.25 (0.05) | 1.5×10^-6^ | 0.0289 |  |
| cg19715081 | *CDK5RAP3* | 17:46048395 | TSS200 | Island | 0.29 (0.06) | 1.9×10^-6^ | 0.0343 |  |
| cg22830707 | *HOXC13* | 12:54332181 | TSS1500 | N_Shore | -0.03 (0.01) | 1.9×10^-6^ | 0.0343 |  |
| cg11078433 | *SLC25A13* | 7:95951561 | TSS200 | Island | 0.03 (0.01) | 2.2×10^-6^ | 0.0365 |  |
| cg05064665 | *PNMT* | 17:37824233 | TSS1500 | Island | 0.27 (0.06) | 2.5×10^-6^ | 0.0365 |  |
| cg04680746 | *NACAD* | 7:45128769 | TSS1500 | Island | 0.30 (0.07) | 2.5×10^-6^ | 0.0365 |  |
| cg13190531 | *POLR3B* | 12:106751832 | TSS200; Body | Island | 0.39 (0.09) | 2.6×10^-6^ | 0.0365 |  |
| cg24776326 | *IRX4* | 5:2206818 |  | OpenSea | -0.05 (0.01) | 2.4×10^-6^ | 0.0365 |  |
| cg07576517 | *SDCCAG8; [CEP170]* | 1:243419299 | TSS200; [TSS1500] | S_Shore | 0.16 (0.04) | 2.7×10^-6^ | 0.0365 |  |
| cg23808931 | *TMEM183A; [TMEM183B]* | 1:202976346 | TSS200; [TSS200] | Island | 0.20 (0.05) | 2.7×10^-6^ | 0.0365 |  |
| cg02376269 | *UBR1* | 15:43398346 | TSS200 | OpenSea | 0.15 (0.03) | 2.9×10^-6^ | 0.0365 |  |
| cg10835423 | *RAP1A* | 1:112162393 | TSS200 | Island | 0.19 (0.04) | 2.9 ×10^-6^ | 0.0365 |  |
| cg03734035 | *NDUFB8* | 10:102289529 | 1stExon | Island | 0.26 (0.06) | 3.4×10^-6^ | 0.0422 |  |
| cg07147063 | *TMEM208; [LRRC29]* | 16:67260764 | TSS1500; [TSS200;5'UTR] | Island | 0.10 (0.02) | 3.5×10^-6^ | 0.0422 |  |
| cg23890800 | *FBRSL1* | 12:133066061 | TSS1500 | Island | 0.24 (0.05) | 3.8×10^-6^ | 0.0449 |  |
| Model is adjusted for maternal age, race/ethnicity, pre-pregnancy BMI, education, job status, gestational age, parity, perceived stress, methylation PCs, genotype PCs, and surrogate variable.  FDR – false discovery rate; LogFC – logarithm of fold change; S.E. – standard error | | | | | | | | |

| **Table S5: Sensitivity analysis for CpGs in placenta associated with level of social support during pregnancies with male fetus adjusted for estimated cell composition^1^ (n=152)** | | | | | | | | | | |
| --- | --- | --- | --- | --- | --- | --- | --- | --- | --- | --- |
| CpG | Gene | Chr: Position | Relation to Gene | Relation to Island | Methylation LogFC (S.E)^1^ | P-value^1^ | P_FDR_^1^ | Methylation LogFC (S.E)^2^ | P- value^2^ | P_FDR_^2^ |
| cg11364468 | *VGF* | 7:100807505 | Body | Island | 0.20 (0.04) | 2.6×10^-8^ | 1.4×10^-7^ | 0.22 (0.03) | 2.9×10^-10^ | 2.6×10^-9^ |
| cg00985086 | *MCTP1* | 5:94620134 | 1stExon | Island | 0.21 (0.04) | 3.2×10^-8^ | 1.4×10^-7^ | 0.17 (0.03) | 1.1×10^-7^ | 5.2×10^-7^ |
| cg03215315 | *GSTCD; INTS12* | 4:106629983 | 1stExon;5'UTR; [TSS200] | Island | 0.18 (0.03) | 2.0×10^-7^ | 6.0×10^-7^ | 0.16 (0.03) | 3.2×10^-6^ | 4.7×10^-6^ |
| cg23797252 | *KNDC1* | 10:135014804 | Body | Island | 0.02 (0.005) | 1.2×10^-5^ | 1.2×10^-5^ | 0.02 (0.005) | 4.4×10^-5^ | 4.9×10^-5^ |
| cg14065446 | *FIBCD1* | 9:133818825 |  | S_Shelf | 0.04 (0.01) | 1.1×10^-6^ | 2.0×10^-6^ | 0.03 (0.008) | 1.1×10^-4^ | 1.1×10^-4^ |
| cg00140191 | *FKBP5* | 6:35656242 | 5'UTR | Island | 0.21 (0.04) | 7.3×10^-7^ | 1.6×10^-6^ | 0.21 (0.04) | 2.1×10^-7^ | 6.2×10^-7^ |
| cg16680530 | *TATDN1; NDUFB9* | 8:125551329 | TSS200; [TSS200] | Island | 0.21 (0.04) | 1.5×10^-6^ | 2.0×10^-6^ | 0.21 (0.04) | 2.8×10^-7^ | 6.2×10^-7^ |
| cg24807054 | *SFRS18* | 6:99873402 | TSS200 | S_Shore | 0.15 (0.03) | 1.4×10^-6^ | 2.0×10^-6^ | 0.14 (0.03) | 3.0×10^-6^ | 4.7×10^-6^ |
| cg18350520 | *KIAA0664* | 17:2595812 | Body | Island | 0.03 (0.006) | 9.5×10^-6^ | 1.1×10^-5^ | 0.02 (0.006) | 4.0×10^-5^ | 4.9×10^-5^ |
| ^1^ Model adjusted for maternal age, race/ethnicity, pre-pregnancy BMI, education, job status, gestational age, parity, perceived stress, methylation PCs, genotype PCs, surrogate variable and estimated cell composition.  ^2^ Model is adjusted for perceived stress, methylation PCs, genotype PCs, surrogate variable.  FDR – false discovery rate; LogFC – logarithm of fold change; S.E – standard error | | | | | | | | | | |

| **Table S6: Sensitivity analysis for CpGs in placenta associated with level of social support during pregnancies with female fetus adjusted for estimated cell composition^1^ (n=149)** | | | | | | | | | | |
| --- | --- | --- | --- | --- | --- | --- | --- | --- | --- | --- |
| CpG | Gene | Chr: Position | Relation to Gene | Relation to Island | Methylation LogFC (S.E)^1^ | P-value^1^ | P_FDR_^1^ | Methylation LogFC (S.E)^2^ | P- value^2^ | P_FDR_^2^ |
| cg04879876 | *ZFP36L2; LOC100129726* | 2:43453558 | 1stExon;5'UTR; TSS1500 | Island | 0.28 (0.04) | 5.8×10^-12^ | 2.0×10^-10^ | 0.26 (0.04) | 3.2×10^-11^ | 1.1×10^-9^ |
| cg16661579 | *C10orf4* | 10:95462206 | 5'UTR;1stExon | Island | 0.32 (0.06) | 1.3×10^-8^ | 1.5×10^-7^ | 0.32 (0.06) | 1.7×10^-8^ | 1.2×10^-7^ |
| cg04777683 | *IVNS1ABP* | 1:185286462 | TSS200 | Island | 0.32 (0.05) | 1.1×10^-9^ | 1.8×10^-8^ | 0.30 (0.06) | 2.1×10^-8^ | 1.2×10^-7^ |
| cg25928819 | *AK055957* | 12:133484026 |  | N_Shore | -0.14 (0.03) | 2.9×10^-7^ | 1.5×10^-6^ | -0.14 (0.03) | 7.6×10^-8^ | 3.4×10^-7^ |
| cg02672368 | *ARHGEF7* | 13:111805930 | Body; TSS200 | Island | 0.21(0.04) | 1.1×10^-7^ | 9.4×10^-7^ | 0.23(0.04) | 1.6×10^-9^ | 2.7×10^-8^ |
| cg03432641 | *SPATS2* | 12:49760606 | TSS200 | Island | 0.13 (0.03) | 7.2×10^-6^ | 8.3×10^-6^ | 0.15 (0.03) | 1.4×10^-7^ | 4.6×10^-7^ |
| cg23065793 | *LOC10012816; SEC62* | 3:169684395 | Body; TSS200 | Island | 0.16 (0.03) | 3.8×10^-7^ | 1.5×10^-6^ | 0.16 (0.03) | 5.7×10^-7^ | 1.3×10^-6^ |
| cg25861327 | *NUSAP1; OIP5* | 15:41625136 | 1stExon;5'UTR; TSS1500 | Island | 0.33 (0.07) | 1.2×10^-6^ | 2.2×10^-6^ | 0.35 (0.07) | 7.7×10^-8^ | 3.4×10^-7^ |
| cg11149743 | *HOXB7* | 17:46688419 | TSS200 | Island | 0.21 (0.04) | 4.4×10^-7^ | 7.1×10^-6^ | 0.19 (0.04) | 5.9×10^-6^ | 8.4×10^-6^ |
| cg10038542 | *ENTPD4* | 8:23315318 | TSS200 | Island | 0.13 (0.03) | 3.9×10^-7^ | 1.5×10^-6^ | 0.12 (0.03) | 6.6×10^-7^ | 1.4×10^-6^ |
| cg24737639 | *NUP37; C12orf48* | 12:102513781 | TSS1500; TSS200 | Island | 0.25 (0.05) | 1.6×10^-7^ | 1.1×10^-6^ | 0.24 (0.05) | 1.2×10^-7^ | 4.9×10^-7^ |
| cg19714762 | *ABHD11* | 7:73153162 | 1stExon; Body; 5'UTR | Island | 0.44 (0.09) | 1.8×10^-7^ | 1.1×10^-6^ | 0.46 (0.08) | 7.5×10^-9^ | 8.8×10^-8^ |
| cg21490179 | *ENTPD3-AS1* | 3:40494492 |  | Island | 0.03 (0.006) | 5.2×10^-7^ | 1.7×10^-6^ | 0.03 (0.007) | 3.3×10^-7^ | 8.8×10^-7^ |
| cg01952989 | *MAD1L1* | 7:1986334 | Body | OpenSea | 0.25 (0.05) | 1.6×10^-6^ | 2.6×10^-6^ | 0.25 (0.05) | 9.9×10^-7^ | 2.0×10^-6^ |
| cg06459916 | *KRCC1* | 2:88355002 | 5'UTR | Island | 0.14 (0.03) | 1.0×10^-6^ | 2.2×10^-6^ | 0.16 (0.03) | 1.1×10^-8^ | 9.9×10^-8^ |
| cg26687565 | *MAML3* | 4:141072046 | Body | Island | 0.15 (0.03) | 1.4×10^-6^ | 2.3×10^-6^ | 0.15 (0.03) | 1.4×10^-6^ | 2.6×10^-6^ |
| cg04484842 | *MYO9A; SENP8* | 15:72410733 | TSS1500; 1stExon; 5'UTR | Island | 0.08 (0.02) | 1.0×10^-6^ | 2.2×10^-6^ | 0.08 (0.02) | 1.3×10^-7^ | 4.5×10^-7^ |
| cg25585364 | *INSIG2* | 2:118846169 | 5'UTR | Island | 0.09 (0.02) | 1.1×10^-6^ | 2.2×10^-6^ | 0.08 (0.02) | 1.5×10^-5^ | 1.7×10^-5^ |
| cg22548088 | *MLLT1* | 19:6271786 | Body | N_Shore | 0.04 (0.01) | 7.5×10^-6^ | 8.4×10^-6^ | 0.04 (0.01) | 1.2×10^-5^ | 1.5×10^-5^ |
| cg09062638 | *C1QB* | 1:23003490 |  | Island | 0.24 (0.05) | 2.9×10^-6^ | 4.0×10^-6^ | 0.23 (0.05) | 5.7×10^-6^ | 8.3×10^-6^ |
| cg08130668 | *C2orf73* | 2:54557891 | TSS200 | Island | 0.25 (0.05) | 1.1×10^-6^ | 2.2×10^-6^ | 0.22 (0.05) | 2.5×10^-6^ | 4.1×10^-6^ |
| cg19715081 | *CDK5RAP3* | 17:46048395 | TSS200 | Island | 0.29 (0.06) | 1.3×10^-6^ | 2.3×10^-6^ | 0.27 (0.06) | 8.5×10^-6^ | 1.1×10^-5^ |
| cg22830707 | *HOXC13* | 12:54332181 | TSS1500 | N_Shore | -0.03 (0.007) | 8.2×10^-7^ | 2.1×10^-6^ | -0.03 (0.007) | 1.1×10^-5^ | 1.4×10^-5^ |
| cg11078433 | *SLC25A13* | 7:95951561 | TSS200 | Island | 0.03 (0.01) | 8.0×10^-7^ | 2.1×10^-6^ | 0.03 (0.01) | 1.6×10^-6^ | 2.8×10^-6^ |
| cg05064665 | *PNMT* | 17:37824233 | TSS1500 | Island | 0.25 (0.06) | 2.9×10^-5^ | 2.9×10^-5^ | 0.25 (0.06) | 8.4×10^-6^ | 1.1×10^-5^ |
| cg04680746 | *NACAD* | 7:45128769 | TSS1500 | Island | 0.31 (0.07) | 3.9×10^-6^ | 5.0×10^-6^ | 0.33 (0.07) | 5.2×10^-7^ | 1.3×10^-6^ |
| cg13190531 | *POLR3B* | 12:106751832 | TSS200; Body | Island | 0.39 (0.09) | 1.9×10^-6^ | 2.9×10^-6^ | 0.41 (0.08) | 3.2×10^-7^ | 8.8×10^-7^ |
| cg24776326 | *IRX4* | 5:2206818 |  | OpenSea | -0.05 (0.01) | 2.0×10^-6^ | 2.9×10^-6^ | -0.05 (0.01) | 3.1×10^-6^ | 4.9×10^-6^ |
| cg07576517 | *SDCCAG8; CEP170* | 1:243419299 | TSS200; TSS1500 | S_Shore | 0.16 (0.04) | 2.6×10^-6^ | 3.7×10^-6^ | 0.17 (0.04) | 1.1×10^-6^ | 2.2×10^-6^ |
| cg23808931 | *TMEM183A; TMEM183B* | 1:202976346 | TSS200; TSS200 | Island | 0.19 (0.05) | 1.0×10^-5^ | 1.1×10^-5^ | 0.16 (0.04) | 5.8×10^-5^ | 5.8×10^-5^ |
| cg02376269 | *UBR1* | 15:43398346 | TSS200 | OpenSea | 0.14 (0.03) | 1.4×10^-5^ | 1.4×10^-5^ | 0.13 (0.03) | 2.4×10^-5^ | 2.7×10^-5^ |
| cg10835423 | *RAP1A* | 1:112162393 | TSS200 | Island | 0.19 (0.04) | 9.1×10^-6^ | 9.9×10^-6^ | 0.16 (0.04) | 3.1×10^-5^ | 3.3×10^-5^ |
| cg03734035 | *NDUFB8* | 10:102289529 | 1stExon | Island | 0.28 (0.06) | 5.8×10^-7^ | 1.7×10^-6^ | 0.24 (0.06) | 5.7×10^-5^ | 5.8×10^-5^ |
| cg07147063 | *TMEM208;*  *LRRC29* | 16:67260764 | TSS1500; TSS200;5'UTR | Island | 0.10 (0.02) | 5.1×10^-6^ | 6.2×10^-6^ | 0.09 (0.02) | 3.3×10^-5^ | 3.6×10^-5^ |
| cg23890800 | *FBRSL1* | 12:133066061 | TSS1500 | Island | 0.24 (0.06) | 5.1×10^-6^ | 6.2×10^-6^ | 0.25 (0.06) | 4.4×10^-6^ | 6.7×10^-6^ |
| ^1^ Model adjusted for maternal age, race/ethnicity, pre-pregnancy BMI, education, job status, gestational age, parity, perceived stress, methylation PCs, genotype PCs, surrogate variable, and estimated cell composition.  ^2^ Model is adjusted for perceived stress, methylation PCs, genotype PCs, surrogate variable.  FDR – false discovery rate; LogFC – logarithm of fold change; S.E – standard error | | | | | | | | | | |
