# Supplementary material for: Prenatal social support in low-risk pregnancy shapes placental epigenome
Source: BMC Med. 2023 Jan 8;21:12. doi: 10.1186/s12916-022-02701-w (PMC9827682; doi:10.1186/s12916-022-02701-w)
Supplement: Supplementary file 4 — Additional file 4: Table S28. Ingenuity canonical pathways of genes annotated to the top 100 CpGs in placenta associated with maternal social support in pregnancies with male fetus. Table S29. Ingenuity canonical pathways of genes annotated to the top 100 CpGs in placenta associated with maternal social support in pregnancies with female fetus. [file 12916_2022_2701_MOESM4_ESM.docx]

| **Table S28: Ingenuity Canonical pathways of genes annotated to the top 100 social support associated CpGs in placenta of male pregnancies. (n=152)** | | | |
| --- | --- | --- | --- |
| **Ingenuity Canonical Pathways** | **log P-value** | **Ratio** | **Molecules** |
| Th2 Pathway | 2.60 | 0.0292 | *ACVR2A, JAK3, PIK3CA, STAT4* |
| JAK/STAT Signaling | 2.32 | 0.0366 | *JAK3, PIK3CA, STAT4* |
| HIPPO signaling | 2.28 | 0.0353 | *LATS2, RASSF1, YWHAH* |
| Th1 and Th2 Activation Pathway | 2.24 | 0.0233 | *ACVR2A, JAK3, PIK3CA, STAT4* |
| IL-9 Signaling | 2.04 | 0.0571 | *JAK3, PIK3CA* |
| Pyrimidine Ribonucleotides Interconversion | 2.04 | 0.0571 | *DHX9, ENTPD4* |
| PD-1, PD-L1 cancer immunotherapy pathway | 2.01 | 0.0283 | *JAK3, LATS2, PIK3CA* |
| Pyrimidine Ribonucleotides De Novo Biosynthesis | 1.99 | 0.0541 | *DHX9, ENTPD4* |
| Oxidative Phosphorylation | 1.96 | 0.027 | *ATP5ME, CYC1, NDUFB9* |
| Kinetochore Metaphase Signaling Pathway | 1.96 | 0.027 | *CCNB1, MAD1L1, SKA1* |
| Role of NANOG in Mammalian Embryonic Stem Cell Pluripotency | 1.87 | 0.025 | *BMP1, JAK3, PIK3CA* |
| Th1 Pathway | 1.85 | 0.0246 | *JAK3, PIK3CA, STAT4* |
| IL-23 Signaling Pathway | 1.81 | 0.0435 | *PIK3CA, STAT4* |
| Cell Cycle: G2/M DNA Damage Checkpoint Regulation | 1.74 | 0.04 | *CCNB1, YWHAH* |
| Sperm Motility | 1.67 | 0.0157 | *AATK, JAK3, PDE4C, PRKG1* |
| Lymphotoxin β Receptor Signaling | 1.66 | 0.0364 | *CYC1, PIK3CA* |
| Semaphorin Neuronal Repulsive Signaling Pathway | 1.61 | 0.0199 | *PDE4C, PIK3CA, PRKG1* |
| IL-2 Signaling | 1.58 | 0.0328 | *JAK3, PIK3CA* |
| Estrogen Receptor Signaling | 1.57 | 0.0123 | *CYC1, JAK3, MED18, NDUFB9, PIK3CA* |
| Epithelial Adherens Junction Signaling | 1.56 | 0.0191 | *ACVR2A, LATS2, YWHAH* |
| ERB2-ERBB3 Signaling | 1.53 | 0.0308 | *JAK3, PIK3CA* |
| CD40 Signaling | 1.5 | 0.0299 | *JAK3, PIK3CA* |
| IL-17A Signaling in Airway Cells | 1.5 | 0.0299 | *JAK3, PIK3CA* |
| Role of JAK1 and JAK3 in γc Cytokine Signaling | 1.48 | 0.029 | *JAK3, PIK3CA* |
| SPINK1 General Cancer Pathway | 1.48 | 0.029 | *JAK3, PIK3CA* |
| Glucocorticoid Receptor Signaling | 1.47 | 0.0103 | *CYC1, FKBP5, JAK3, NDUFB9, PIK3CA, YWHAH* |
| Mitochondrial Dysfunction | 1.47 | 0.0175 | *ATP5ME, CYC1, NDUFB9* |
| IL-7 Signaling Pathway | 1.38 | 0.0256 | *JAK3, PIK3CA* |
| FLT3 Signaling in Hematopoietic Progenitor Cells | 1.36 | 0.025 | *PIK3CA, STAT4* |
| Regulation of the Epithelial Mesenchymal Transition by Growth Factors Pathway | 1.34 | 0.0156 | *JAK3, LATS2, PIK3CA* |
| PDGF Signaling | 1.31 | 0.0233 | *JAK3, PIK3CA* |

| **Table S29: Ingenuity Canonical pathways of genes annotated to the top 100 social support associated CpGs in placenta of female pregnancies. (n=149)** | | | |
| --- | --- | --- | --- |
| **Ingenuity Canonical Pathways** | **Log**  **P-value** | **Ratio** | **Molecules** |
| Neuregulin Signaling | 2.74 | 0.0342 | *ITGA3, PRKCZ, RAP1A, RPS6KB2* |
| RAC Signaling | 2.48 | 0.029 | *ANK1, ITGA3, PRKCZ, RAP1A* |
| HER-2 Signaling in Breast Cancer | 2.47 | 0.022 | *NUP37, PRKCZ, RAP1A, RPS6KB2, RPTOR* |
| Insulin Receptor Signaling | 2.45 | 0.0286 | *PRKCZ, RAP1A, RPS6KB2, RPTOR* |
| PTEN Signaling | 2.35 | 0.0267 | *ITGA3, PRKCZ, RAP1A, RPS6KB2* |
| NF-κB Activation by Viruses | 2.3 | 0.0385 | *ITGA3, PRKCZ, RAP1A* |
| Inhibition of ARE-Mediated mRNA Degradation Pathway | 2.24 | 0.0248 | *AGO3, EXOSC2, PSMB9, ZFP36L2* |
| IL-4 Signaling | 2.09 | 0.0323 | *NFATC4, RAP1A, RPS6KB2* |
| Regulation of eIF4 and p70S6K Signaling | 2.08 | 0.0223 | *AGO3, ITGA3, PRKCZ, RAP1A* |
| Endothelin-1 Signaling | 1.97 | 0.0208 | *ECE1, PNPLA3, PRKCZ, RAP1A* |
| Virus Entry via Endocytic Pathways | 1.95 | 0.0288 | *AP2S1, PRKCZ, RAP1A* |
| IGF-1 Signaling | 1.95 | 0.0288 | *PRKCZ, RAP1A, RPS6KB2* |
| Paxillin Signaling | 1.91 | 0.0278 | *ARHGEF7, ITGA3, RAP1A* |
| Antigen Presentation Pathway | 1.89 | 0.0513 | *PSMB9, TAP1* |
| mTOR Signaling | 1.83 | 0.0189 | *PRKCZ, RAP1A, RPS6KB2, RPTOR* |
| FAK Signaling | 1.82 | 0.0256 | *ARHGEF7, ITGA3, RAP1A* |
| PAK Signaling | 1.81 | 0.0254 | *ARHGEF7, ITGA3, RAP1A* |
| NGF Signaling | 1.81 | 0.0254 | *PRKCZ, RAP1A, RPS6KB2* |
| 14-3-3-mediated Signaling | 1.72 | 0.0236 | *PRKCZ, RAP1A, TUBB* |
| fMLP Signaling in Neutrophils | 1.69 | 0.0229 | *NFATC4, PRKCZ, RAP1A* |
| HGF Signaling | 1.68 | 0.0227 | *ITGA3, PRKCZ, RAP1A* |
| UVC-Induced MAPK Signaling | 1.67 | 0.0392 | *PRKCZ, RAP1A* |
| Apelin Endothelial Signaling Pathway | 1.61 | 0.0213 | *PRKCZ, RAP1A, RPS6KB2* |
| Axonal Guidance Signaling | 1.59 | 0.0118 | *ARHGEF7, ITGA3, NFATC4, PRKCZ, RAP1A, TUBB* |
| PI3K Signaling in B Lymphocytes | 1.59 | 0.021 | *NFATC4, PRKCZ, RAP1A* |
| CNTF Signaling | 1.58 | 0.0351 | *RAP1A, RPS6KB2* |
| Endometrial Cancer Signaling | 1.54 | 0.0333 | *MLH1, RAP1A* |
| Thrombopoietin Signaling | 1.5 | 0.0317 | *PRKCZ, RAP1A* |
| Insulin Secretion Signaling Pathway | 1.49 | 0.0148 | *AGO3, PRKCZ, RAP1A, RPS6KB2* |
| Ovarian Cancer Signaling | 1.48 | 0.019 | *MLH1, RAP1A, RPS6KB2* |
| Cardiac Hypertrophy Signaling (Enhanced) | 1.47 | 0.0111 | *ACVR2B, ITGA3, NFATC4, PRKCZ, RAP1A, RPS6KB2* |
| Protein Ubiquitination Pathway | 1.47 | 0.0145 | *DNAJC10, PSMB9, TAP1, UBR1* |
| Opioid Signaling Pathway | 1.45 | 0.0144 | *AP2S1, PRKCZ, RAP1A, RPS6KB2* |
| ERBB4 Signaling | 1.44 | 0.0294 | *PRKCZ, RAP1A* |
| Agrin Interactions at Neuromuscular Junction | 1.41 | 0.0286 | *ARHGEF7, RAP1A* |
| Hepatic Fibrosis Signaling Pathway | 1.41 | 0.0119 | *ACVR2B, ITGA3, PRKCZ, RAP1A, RPS6KB2* |
| Growth Hormone Signaling | 1.4 | 0.0282 | *PRKCZ, RPS6KB2* |
| Germ Cell-Sertoli Cell Junction Signaling | 1.39 | 0.0175 | *ITGA3, RAP1A, TUBB* |
| ERK5 Signaling | 1.39 | 0.0278 | *RAP1A, RPS6KB2* |
| Senescence Pathway | 1.37 | 0.0135 | *ACVR2B, NFATC4, PRKCZ, RAP1A* |
| Macropinocytosis Signaling | 1.35 | 0.0263 | *PRKCZ, RAP1A* |
| VDR/RXR Activation | 1.33 | 0.0256 | *PRKCZ, YY1* |
| IL-3 Signaling | 1.32 | 0.0253 | *PRKCZ, RAP1A* |
